# Supplementary material for: c-Src functionality controls self-renewal and glucose metabolism in MCF7 breast cancer stem cells
Source: PLoS One. 2020 Jul 16;15(7):e0235850. doi: 10.1371/journal.pone.0235850 (PMC7365443; doi:10.1371/journal.pone.0235850)
Supplement: S1 File — (PDF) [file pone.0235850.s001.pdf]

c-Src functionality controls self-renewal, tumorigenicity and glucose metabolism in breast cancer stem cells

Víctor Mayoral-Varo[1*], Annarica Calcabrini[1,2*], María Pilar Sánchez-Bailón[1,3*], Óscar H. Martínez-Costa[1], Cristina González-Páramos[1], Sergio Ciordia[4], David Hardisson[5,6], Juan J. Aragón[1], Miguel Ángel Fernández-Moreno[1,7], and Jorge Martín-Pérez[1,6,‡]

Supplementary Materials:

1. Supplementary Methods: 1.1. Gene Set Enrichment Analyses (GSEA); 1.2. RNA preparation, qRT-PCR of c-Myc and HIF-1; 1.3. Detection of mitochondrial superoxide and reactive oxygen species (ROS).

2. Supplementary figures and tables: 2.1. Figure S1. Flow cytometric analysis of isotypic immunoglobulin labeling. **2.2. Figure S2.** Three generations of mammospheres derived from MCF7-Tet-On-SrcDN. **2.3. Figure S3.** Flow cytometric analysis of CD24-PE and CD44-APC markers in control and Doxy-treated (2 μg/ml) 3rd generation mammospheres. **2.4. Figure S4.** Quantitative proteomic data from CD24^{-} derived from MCF7-Tet-On-SrcDN. **2.5. Figure S5**. Gene set enrichment analysis (GSEA) of mammospheres derived from MCF7-Tet-On-c-SrcDN cells. **2.6. Figure S6**. c-Myc and HIF-1 expression. **2.7. Figure S7**. Profiles of oxygen consumption rates of mammospheres cells derived from MCF7-Tet-On-SrcDN. **2.8. Figure S8.** Detection of MitoSOX and ROS in CD24^{-} cells derived from MCF7-Tet-On-SrcDN. **2.9.** List of Abbreviations. **2.10. Table S1.** Summary of quantitated proteomic analyses. **2.11. Table S2.** Antibodies employed in these studies. **2.12.** Reports of MCF7 cell line authentication. **2.13.** Figures S9, S10, S11, and S12. Uncropped scan of the western blots of the manuscript. **2.14.** Quantitation of WB.

3. References

Supplementary Materials:

1. Supplementary Methods

1.1. Gene Set Enrichment Analyses (GSEA)

The results of the quantitative proteomics analysis of BCSCs derived from MCF7-Tet-On-c-SrcDN showed a series of proteins whose levels were altered by the expression of c-SrcDN. These proteins were analyzed by GSEA, a computational method that determines whether a set of proteins/genes presents statistically significant differences between two samples.

1.2.RNA preparation, qRT-PCR of c-Myc and Hif-1

RNA was isolated from three independent experiments performed in triplicate using RNeasy kit (Qiagen). After testing for RNA integrity, triplicate RNAs from each experiment were pooled, then 1 μg of the purified RNAs was employed to synthesize the corresponding cDNAs using the Maxima First Strand cDNA Synthesis Kit for RT-qPCR with dsDNase (Thermo Fisher Scientific), followed by SYBR green (PowerUp™ SYBR™ Green Master Mix, Thermo Fisher Scientific)

RTqPCR using an Applied Biosystems StepOnePlus™ real-time thermocycler (Thermo Fisher Scientific). Cycle thresholds were normalized to β-actin levels. Expression of c-Myc and HIF-1 was determined using the following primers: c-Myc, forward 5'-CCCGCTTCTCTGAAAGGCTC TC-3', reverse 5'-CTCTGCTGCTGCTGCTGGTAG-3'; HIF-1, forward: 5'-TTGATGGGATATG AGCCAGA-3', reverse: 5'-TGTCCTGTGGTGACTTGTCC-3'; β-actin (employed to normalize), forward 5´-GCGAGCACAGAGCCTCGCCTT-3', reverse 5´-CATCATCCATGGTGAGCTGGCG G-3'. Relative expression of c-Myc and HIF-1 was calculated as the difference in cycle threshold (ΔCt) between target gene and β-actin respectively; ΔΔCt was the difference between ΔCt values of test sample and that of control. Relative expression of target genes was calculated as $2^{-\Delta\Delta Ct}$.

1.3. Detection of mitochondrial superoxide and reactive oxygen species (ROS)

Determination of Mitochondrial superoxide and ROS in CD24-neg cells derived from MCF7-Tet-ON-SrcDN in absence or presence of Doxy (2 μg/mL) was carried out employing fluorescence dies, MitoSOX red (Invitrogen, M36008), and DCFH-DA (abcam, ab113851), as described [1]. In parallel, from a portion of the same samples, the expression levels of catalase, and manganese superoxide dismutase (MnSOD) were determined by immunoblotting.

2. Supplementary figures and tables

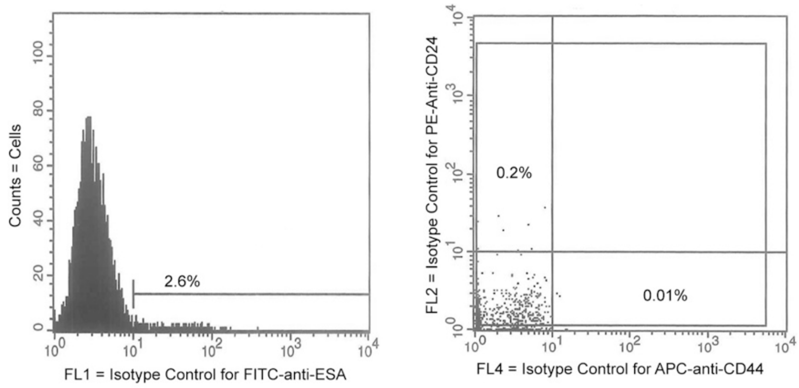

2.1. Figure S1. Flow cytometric analysis of isotypic immunoglobulin labelling. Cells were incubated with FITC-mouse IgG1 (isotypic control for ESA staining, histogram on the left), with PE-mouse IgG2a (isotypic control for CD24 labelling) and APC-mouse IgG2b (isotypic control for CD44 labelling), dot plot on the right. The percentage of positive cells are indicated. Results from one representative experiment.

Three generations of Mammospheres from MCF7-Tet-On-SrcDN

	1st generation		2nd generation		3rd generation	
	- Doxy	+ Doxy	- Doxy	+ Doxy	- Doxy	+ Doxy
Exp. 1	7.76	2.84	9.99	5.16	10.40	6.70
Exp. 2	4.99	3.44	7.95	5.43	10.80	8.80
Exp. 3	5.73	3.73	6.50	4.20	10.30	8.10
Exp. 4	8.56	4.87	8.25	6.44	9.60	3.50
Exp. 5	6.60	4.00	10.50	7.00	8.20	3.90
Mean	6.73	3.78	8.64	5.65	9.86	6.21
SD	1.5	1.0	1.6	1.0	1.0	2.0
t-Student	0.004		0.009		0.014	
	p<0.01 **		p<0.01 **		p<0.5 *	

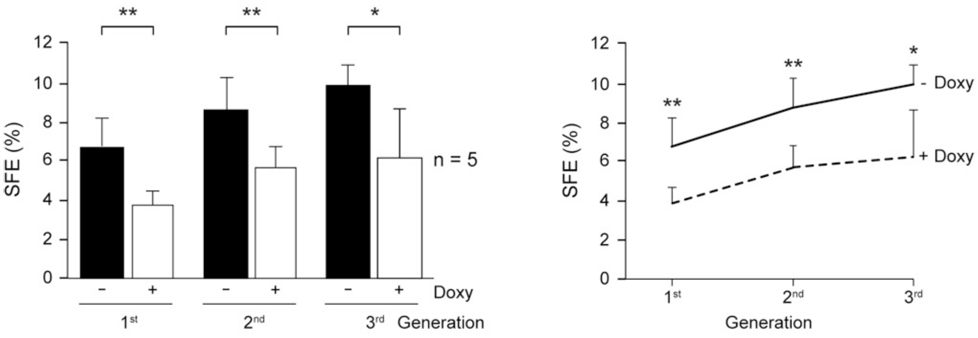

Figure S2

2.2. Figure S2. Three generations of mammospheres derived from MCF7-Tet-On-SrcDN. Cells were initially plated in 6-well ultralow attachment plates at $2x10^3$ cells/well in mammosphere culture media without Doxy. Fifteen days later, mammospheres were dissociated into single cells

that were plated in 6-well ultralow attachment plates at $2x10^3$ cells/well in mammosphere culture media, 3 wells without Doxy (Control) and 3 wells with Doxy (2 μg/ml). These two groups were maintained during the three generations, as well as during the re-plating (see Materials and methods). All experiments were carried out in triplicates. Values represent the Mean+/-SD, n=5.

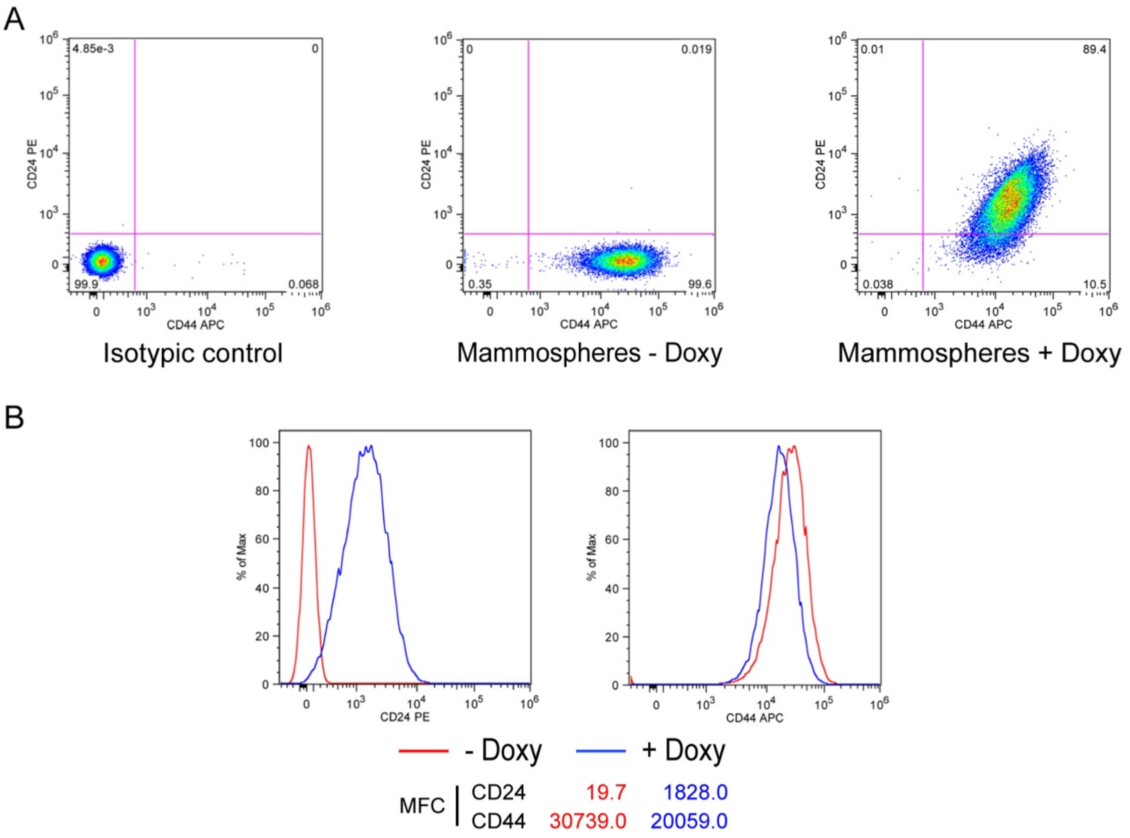

FIGURE S3. Flow cytometric analysis of CD24-PE and CD44-APC markers in control and Doxy-treated (2 μg/ml) 3rd generation mammospheres. (A) Cells obtained from dissociated mammospheres were incubated with PE-mouse IgG2a and APC-mouse IgG2b immunoglobulins (dot plot on the left, isotypic control) or with anti-CD24-PE and anti-CD44-APC monoclonal antibodies (dot plots on the right, Mammospheres -/+ Doxy). The percentage of cells in each quadrant is indicated. (B) Histograms showing CD24-PE and CD44-APC labelling in control (red line) and Doxy-treated (blue line) samples. The values of the mean fluorescence channel (MFC) related to CD24 and CD44 expression are reported. Results from one representative experiment.

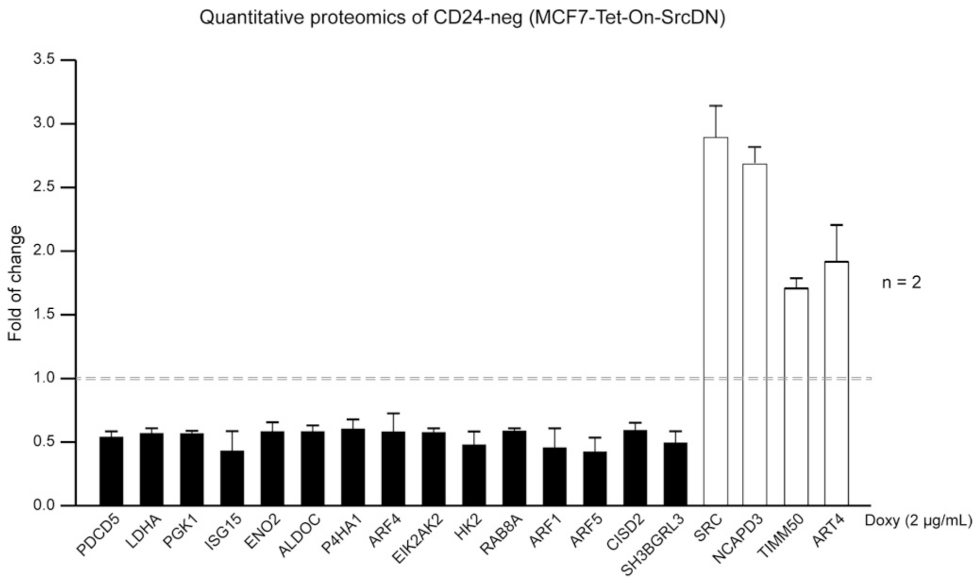

2.4. Figure S4. Quantitative proteomic data from mammospheres derived from MCF7-Tet-On-SrcDN. Graphic of bars from data shown in Table 1, refer to -Doxy considered as 1 (dashed line). Black bars showed proteins whose expression is reduced upon SrcDN induction, open bars represented proteins upregulated by SrcDN expression, showing c-Src increased up to three folds.

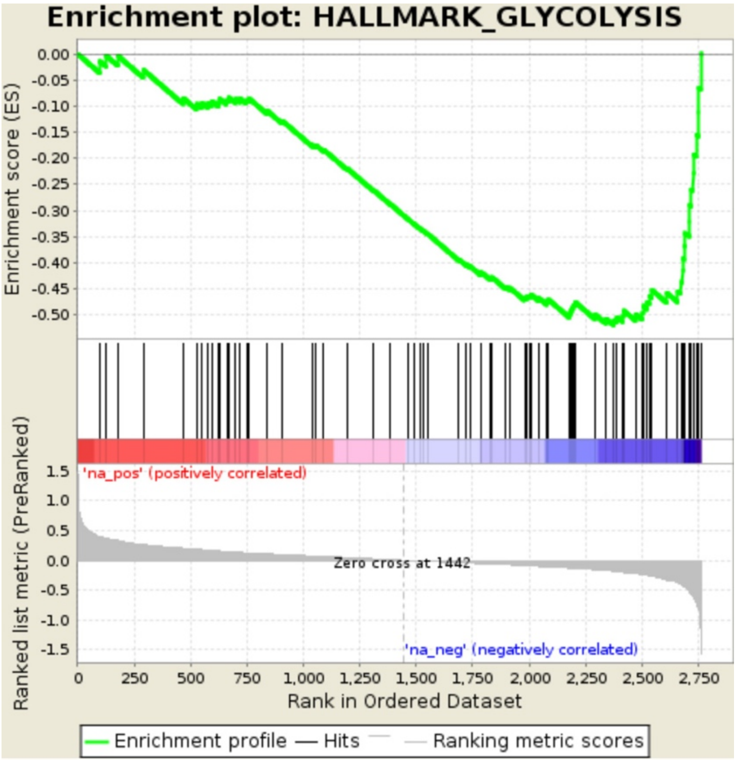

2.5. Figure S5. Gene set enrichment analysis (GSEA) of mammospheres derived from MCF7-Tet-On-c-SrcDN cells. A significant downregulation of proteins involved in glycolysis was detected in mammospheres upon expression of SrcDN.

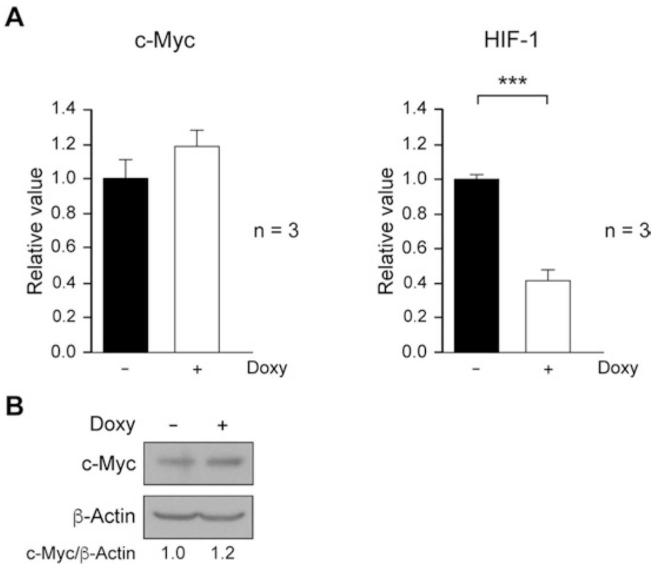

2.6. Figure S6. c-Myc and HIF-1 expression in control and Doxy-treated (2 μg/mL) 3rd mammosphere generation. (**A**) Expression of c-Myc and HIF-1 by SYBR Green q-RT-PCR using β-actin as endogenous gene (see Materials and methods). Results are shown as Mean ± SD of relative levels in three independent experiments, considering arbitrarily -Doxy (Control) as 1 (***$p<0.001$). (**B**) Immunoblotting analysis of c-Myc expression employing β-actin as loading control. Results are referred to -Doxy (Control) considered as 1.

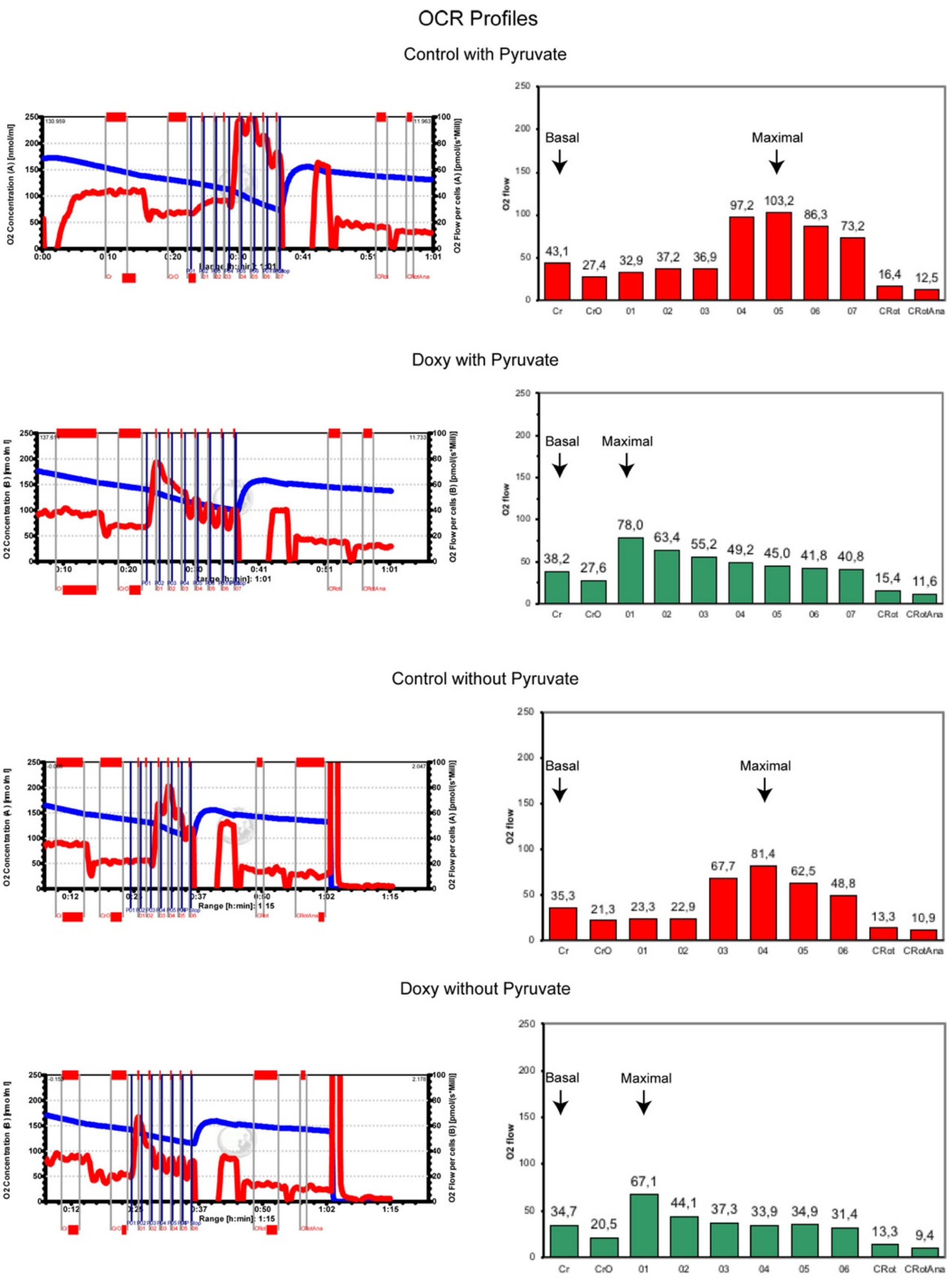

2.7. Figure S7. Profiles of oxygen consumption rates in 3rd generation mammosphere cells derived from MCF7-Tet-On-SrcDN. Experiments were carried out as described in main document. Representative profiles of OCR from mammosphere cells and graphic bars showing Basal and Maximal consumption measured in mammospheres culture media with and without pyruvate.

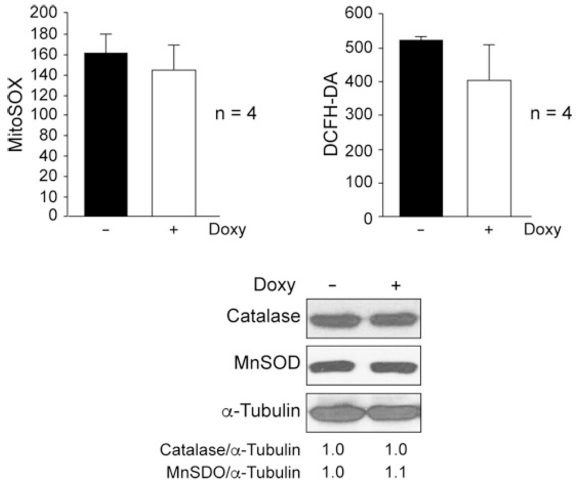

2.8. Figure S8. Detection of MitoSOX and ROS in 3rd generation mammosphere cells derived from MCF7-Tet-On-SrcDN. Mitochondrial superoxide and ROS determined in mammosphere cells derived from MCF7-Tet-ON-SrcDN in absence or presence of Doxy (2 μg/mL) was carried by flow cytometry, were quantified in arbitrary units. From a portion of the same samples, the expression levels of catalase and MnSOD from mammosphere cells was determined by immunoblotting, employing α-Tubulin as a loading control. These are representative results from 4 independent experiments. The ratios refer to -Doxy considered as 1.

c-Src functionality controls self-renewal, tumorigenicity and glucose metabolism in breast cancer stem cells, by V. Mayoral et al.

List of abbreviations:

APC	Allophycocyanin
ATP	Adenosine triphosphate
BCSCs	Breast Cancer Stem Cells
BSA	Bovine Serum Albumin
CSK	C-terminal Src Kinase
CD24-pos	CD24-high or CD24 positive
CD24-neg	CD24-low or CD24 negative
DAPI	4′, 6-diamidino-2-phenylindole
DCFH-DA	Dichloro-dihydro-fluorescein diacetate
DMEM	Dulbecco's Modified Eagle's Medium
Doxy	Doxycycline (analogue of Tetracycline)
EGF	Epidermal Growth Factor
ESA	Epithelial Specific Antigen
FCS	Fetal Calf Serum
FDR	False Discovery Rate
bFGF	basic Fibroblast Growth Factor
FITC	Fluorescein Isothiocyanate
FACS	Fluorescence-Activated Cell Sorting
Fak	Focal adhesion kinase
G6PD	Glucose-6-phosphate-Dehydrogenase
GAPDH	Glyceraldehyde-3-phosphate-Dehydrogenase
Glut-1	Glucose Transporter 1
GSEA	Gene Set Enrichment Analysis
HK	Hexokinase
LDH	Lactate dehydrogenase
MCT-1 (SLC16A1)	Proton coupled monocarboxylate transporter-1
MMP2, MMP7	Metalloproteinase 2 or 7
MnSOD	Manganese-dependent Superoxide Dismutase
MAb	Monoclonal Antibody
NAD	Nicotinamide Adenine Dinucleotide
OCR	Oxygen consumption rate
PARP	Poly (ADP-ribose) polymerases
PBS	Phosphate Buffered Saline
PE	Phycoerythrin
PK	Pyruvate Kinase
ROS	Reactive Oxygen Species
SD	Standard Deviation
SFE	Sphere Formation Efficiency (self-renewal)
SFKs	Src-family tyrosine kinases
shRNA	Short hairpin RNA
SrcDN	Dominant negative variant of c-Src (c-Src-K295M/Y527F)
Tet-On system	Doxycycline-inducible Gene Expression

From Table I

ALDOC	Fructose-bisphosphate aldolase C
ARF1	ADP-ribosylation factor 1
ARF4	ADP-ribosylation factor 4
ARF5	ADP-ribosylation factor 5
ART4	Ecto-ADP-ribosyltransferase 4
CISD2	CDSH iron-sulfur domain-containing protein
EIF2AK2	Interferon-induced, double-stranded RNA-activated protein kinase
ENO2	Gamma-enolase
HK2	Hexokinase-2
ISG15	Ubiquitin-like protein ISG15
LDHA	L-lactate dehydrogenase A chain
NCAPD3	Condensin-2 complex subunit D3
P4HA1	Prolyl 4-hydroxylase subunit alpha-1
PDCD5	Programmed cell death protein 5
PGK1	Phosphoglycerate kinase 1
RAB8A	Ras-related protein Rab-8A
SH3BGRL3	SH3 domain-binding glutamic acid-rich-like protein 3
SRC (c-Src)	Proto-oncogene tyrosine-protein kinase Src
TIMM50	Mitochondrial import inner membrane translocase subunit TIM50

2.9. List of abbreviations

DIFFERENTIAL PROTEINS FOR 5% FDR AT QUANTITATION LEVEL		Doxy-1/Control-1			Doxy-2/Control-2		
Protein_AC	Description	Ratio	Log2	FDR	Ratio	Log2	FDR
A6NFQ2	Protein FAM115C OS=Homo sapiens GN=FAM115C PE=2 SV=2	1,034	0,049	100,28%	0,660	-0,599	1,86%
O14737	Programmed cell death protein 5 OS=Homo sapiens GN=PDCD5 PE=1 SV=3	0,527	-0,923	0,33%	0,576	-0,797	0,06%
O14745	Na(+)/H(+) exchange regulatory cofactor NHE-RF1 OS=Homo sapiens GN=SLC9A3R1 PE=1 SV=4	0,845	-0,244	87,65%	0,619	-0,692	0,42%
O14773	Tripeptidyl-peptidase 1 OS=Homo sapiens GN=TPP1 PE=1 SV=2	1,235	0,304	93,56%	1,620	0,696	3,35%
O43852	Calumenin OS=Homo sapiens GN=CALU PE=1 SV=2	1,248	0,320	91,88%	0,633	-0,659	0,67%
O75506	Heat shock factor-binding protein 1 OS=Homo sapiens GN=HSBP1 PE=1 SV=1	0,504	-0,987	0,14%	0,842	-0,248	70,65%
O94855	Protein transport protein Sec24D OS=Homo sapiens GN=SEC24D PE=1 SV=2	0,906	-0,142	97,19%	0,656	-0,609	1,62%
O95197	Reticulon-3 OS=Homo sapiens GN=RTN3 PE=1 SV=2	0,593	-0,755	2,65%	0,956	-0,065	95,30%
O95400	CD2 antigen cytoplasmic tail-binding protein 2 OS=Homo sapiens GN=CD2BP2 PE=1 SV=1	1,020	0,028	99,71%	0,668	-0,581	2,43%
P00338	L-lactate dehydrogenase A chain OS=Homo sapiens GN=LDHA PE=1 SV=2	0,589	-0,764	2,36%	0,567	-0,818	0,04%
P00441	Superoxide dismutase [Cu-Zn] OS=Homo sapiens GN=SOD1 PE=1 SV=2	1,051	0,072	100,25%	0,603	-0,731	0,20%
P00558	Phosphoglycerate kinase 1 OS=Homo sapiens GN=PGK1 PE=1 SV=3	0,583	-0,777	2,05%	0,569	-0,813	0,04%
P01023	Alpha-2-macroglobulin OS=Homo sapiens GN=A2M PE=1 SV=3	0,880	-0,185	93,98%	0,457	-1,128	0,00%
P02538	Keratin, type II cytoskeletal 6A OS=Homo sapiens GN=KRT6A PE=1 SV=3	2,912	1,542	0,00%	1,101	0,139	98,08%
P02768	Serum albumin OS=Homo sapiens GN=ALB PE=1 SV=2	3,049	1,608	0,00%	1,647	0,720	2,30%
P02792	Ferritin light chain OS=Homo sapiens GN=FTL PE=1 SV=2	1,362	0,446	72,52%	0,588	-0,766	0,09%
P02794	Ferritin heavy chain OS=Homo sapiens GN=FTH1 PE=1 SV=2	0,934	-0,098	97,89%	0,630	-0,666	0,59%
P04264	Keratin, type II cytoskeletal 1 OS=Homo sapiens GN=KRT1 PE=1 SV=6	2,991	1,581	0,00%	0,927	-0,109	94,84%
P04350	Tubulin beta-4A chain OS=Homo sapiens GN=TUBB4A PE=1 SV=2	0,547	-0,871	0,68%	1,280	0,356	76,52%
P05161	Ubiquitin-like protein ISG15 OS=Homo sapiens GN=ISG15 PE=1 SV=5	0,540	-0,889	0,54%	0,337	-1,568	0,00%
P05387	60S acidic ribosomal protein P2 OS=Homo sapiens GN=RPLP2 PE=1 SV=1	0,588	-0,766	2,34%	0,809	-0,307	48,87%
P06703	Protein S100-A6 OS=Homo sapiens GN=S100A6 PE=1 SV=1	0,813	-0,298	76,92%	0,474	-1,076	0,00%
P08779	Keratin, type I cytoskeletal 16 OS=Homo sapiens GN=KRT16 PE=1 SV=4	2,164	1,114	0,13%	1,090	0,124	99,00%
P09104	Gamma-enolase OS=Homo sapiens GN=ENO2 PE=1 SV=3	0,541	-0,885	0,56%	0,642	-0,639	0,94%
P09972	Fructose-bisphosphate aldolase C OS=Homo sapiens GN=ALDOC PE=1 SV=2	0,569	-0,812	1,37%	0,612	-0,708	0,31%
P10412	Histone H1.4 OS=Homo sapiens GN=HIST1H1E PE=1 SV=2	2,303	1,204	0,03%	1,252	0,324	83,43%
P10606	Cytochrome c oxidase subunit 5B, mitochondrial OS=Homo sapiens GN=COX5B PE=1 SV=2	0,765	-0,387	62,42%	0,629	-0,669	0,59%
P10636	Microtubule-associated protein tau OS=Homo sapiens GN=MAPT PE=1 SV=5	1,072	0,100	99,29%	0,662	-0,595	1,93%
P12429	Annexin A3 OS=Homo sapiens GN=ANXA3 PE=1 SV=3	1,970	0,978	0,74%	1,353	0,437	50,49%
P12931	Proto-oncogene tyrosine-protein kinase Src OS=Homo sapiens GN=SRC PE=1 SV=3	3,079	1,622	0,00%	2,738	1,453	0,00%
P13645	Keratin, type I cytoskeletal 10 OS=Homo sapiens GN=KRT10 PE=1 SV=6	2,615	1,387	0,00%	1,497	0,582	14,78%
P13647	Keratin, type II cytoskeletal 5 OS=Homo sapiens GN=KRT5 PE=1 SV=3	3,290	1,718	0,00%	1,383	0,468	41,10%
P13674	Prolyl 4-hydroxylase subunit alpha-1 OS=Homo sapiens GN=P4HA1 PE=1 SV=2	0,572	-0,807	1,41%	0,653	-0,616	1,45%
P15559	NAD(P)H dehydrogenase [quinone] 1 OS=Homo sapiens GN=NQO1 PE=1 SV=1	1,911	0,935	1,36%	1,363	0,447	46,57%
P15941	Mucin-1 OS=Homo sapiens GN=MUC1 PE=1 SV=3	0,719	-0,477	41,20%	0,685	-0,546	4,33%
P16188	HLA class I histocompatibility antigen, A-30 alpha chain OS=Homo sapiens GN=HLA-A PE=1 SV=2	0,762	-0,392	62,05%	0,593	-0,754	0,12%
P16455	Methylated-DNA--protein-cysteine methyltransferase OS=Homo sapiens GN=MGMT PE=1 SV=1	1,006	0,009	99,80%	1,775	0,828	0,41%
P16949	Stathmin OS=Homo sapiens GN=STMN1 PE=1 SV=3	0,800	-0,322	74,31%	0,669	-0,580	2,43%
P17655	Calpain-2 catalytic subunit OS=Homo sapiens GN=CAPN2 PE=1 SV=6	2,078	1,055	0,27%	0,988	-0,018	98,23%
P18085	ADP-ribosylation factor 4 OS=Homo sapiens GN=ARF4 PE=1 SV=3	0,494	-1,017	0,09%	0,684	-0,549	4,15%
P19525	Interferon-induced, double-stranded RNA-activated protein kinase OS=Homo sapiens GN=EIF2AK2 PE=1 SV=2	0,571	-0,807	1,43%	0,594	-0,751	0,13%
P20933	N(4)-(beta-N-acetylglucosaminyl)-L-asparaginase OS=Homo sapiens GN=AGA PE=1 SV=2	2,058	1,042	0,33%	1,388	0,473	40,60%
P30838	Aldehyde dehydrogenase, dimeric NADP-preferring OS=Homo sapiens GN=ALDH3A1 PE=1 SV=3	1,980	0,986	0,68%	1,574	0,655	6,25%
P35527	Keratin, type I cytoskeletal 9 OS=Homo sapiens GN=KRT9 PE=1 SV=3	3,190	1,674	0,00%	0,696	-0,523	6,20%
P35908	Keratin, type II cytoskeletal 2 epidermal OS=Homo sapiens GN=KRT2 PE=1 SV=2	2,641	1,401	0,00%	1,557	0,639	7,66%
P40616	ADP-ribosylation factor-like protein 1 OS=Homo sapiens GN=ARL1 PE=1 SV=1	0,595	-0,750	2,83%	0,777	-0,364	35,74%
P42224	Signal transducer and activator of transcription 1-alpha/beta OS=Homo sapiens GN=STAT1 PE=1 SV=2	0,728	-0,459	45,86%	0,580	-0,785	0,07%
P42695	Condensin-2 complex subunit D3 OS=Homo sapiens GN=NCAPD3 PE=1 SV=2	2,595	1,376	0,00%	2,776	1,473	0,00%

**P43487**	Ran-specific GTPase-activating protein OS=Homo sapiens GN=RANBP1 PE=1 SV=1	0,836	-0,258	84,90%	0,636	-0,653	0,74%
**P45973**	Chromobox protein homolog 5 OS=Homo sapiens GN=CBX5 PE=1 SV=1	0,896	-0,158	97,12%	0,666	-0,586	2,26%
**P49207**	60S ribosomal protein L34 OS=Homo sapiens GN=RPL34 PE=1 SV=3	1,859	0,894	2,05%	1,163	0,218	95,08%
**P51153**	Ras-related protein Rab-13 OS=Homo sapiens GN=RAB13 PE=1 SV=1	0,774	-0,370	65,52%	0,674	-0,570	2,88%
**P51858**	Hepatoma-derived growth factor OS=Homo sapiens GN=HDGF PE=1 SV=1	0,821	-0,285	79,15%	0,452	-1,144	0,00%
**P52789**	Hexokinase-2 OS=Homo sapiens GN=HK2 PE=1 SV=2	0,546	-0,874	0,67%	0,428	-1,226	0,00%
**P54252**	Ataxin-3 OS=Homo sapiens GN=ATXN3 PE=1 SV=4	1,524	0,608	37,92%	1,661	0,732	1,85%
**P56589**	Peroxisomal biogenesis factor 3 OS=Homo sapiens GN=PEX3 PE=1 SV=1	0,588	-0,765	2,33%	0,870	-0,200	79,65%
**P61006**	Ras-related protein Rab-8A OS=Homo sapiens GN=RAB8A PE=1 SV=1	0,585	-0,773	2,14%	0,606	-0,722	0,24%
**P62158**	Calmodulin OS=Homo sapiens GN=CALM1 PE=1 SV=2	1,147	0,197	97,93%	0,586	-0,770	0,09%
**P62330**	ADP-ribosylation factor 6 OS=Homo sapiens GN=ARF6 PE=1 SV=2	0,507	-0,979	0,16%	0,773	-0,371	33,35%
**P68366**	Tubulin alpha-4A chain OS=Homo sapiens GN=TUBA4A PE=1 SV=1	1,818	0,862	3,05%	0,963	-0,054	95,41%
**P80303**	Nucleobindin-2 OS=Homo sapiens GN=NUCB2 PE=1 SV=2	2,128	1,089	0,18%	1,565	0,647	6,88%
**P84077**	ADP-ribosylation factor 1 OS=Homo sapiens GN=ARF1 PE=1 SV=2	0,363	-1,464	0,00%	0,559	-0,838	0,03%
**P84085**	ADP-ribosylation factor 5 OS=Homo sapiens GN=ARF5 PE=1 SV=2	0,365	-1,452	0,00%	0,497	-1,009	0,00%
**Q01650**	Large neutral amino acids transporter small subunit 1 OS=Homo sapiens GN=SLC7A5 PE=1 SV=2	2,081	1,057	0,27%	0,948	-0,077	95,84%
**Q08380**	Galectin-3-binding protein OS=Homo sapiens GN=LGALS3BP PE=1 SV=1	0,631	-0,664	7,92%	0,402	-1,315	0,00%
**Q13185**	Chromobox protein homolog 3 OS=Homo sapiens GN=CBX3 PE=1 SV=4	0,898	-0,155	97,30%	0,681	-0,554	3,83%
**Q13268**	Dehydrogenase/reductase SDR family member 2, mitochondrial OS=Homo sapiens GN=DHRS2 PE=1 SV=4	1,498	0,583	45,23%	0,630	-0,667	0,60%
**Q14244**	Ensconsin OS=Homo sapiens GN=MAP7 PE=1 SV=1	1,864	0,898	2,02%	1,108	0,148	97,92%
**Q14683**	Structural maintenance of chromosomes protein 1A OS=Homo sapiens GN=SMC1A PE=1 SV=2	0,531	-0,912	0,39%	0,919	-0,122	93,63%
**Q14956**	Transmembrane glycoprotein NMB OS=Homo sapiens GN=GPNMB PE=1 SV=2	5,566	2,477	0,00%	1,765	0,820	0,45%
**Q15125**	3-beta-hydroxysteroid-Delta(8),Delta(7)-isomerase OS=Homo sapiens GN=EBP PE=1 SV=3	1,571	0,651	27,03%	0,614	-0,704	0,34%
**Q16881**	Thioredoxin reductase 1, cytoplasmic OS=Homo sapiens GN=TXNRD1 PE=1 SV=3	2,022	1,016	0,47%	1,274	0,349	76,64%
**Q3ZCQ8**	Mitochondrial import inner membrane translocase subunit TIM50 OS=Homo sapiens GN=TIMM50 PE=1 SV=2	1,769	0,823	4,91%	1,673	0,743	1,65%
**Q53HC9**	Protein TSSC1 OS=Homo sapiens GN=TSSC1 PE=1 SV=2	1,805	0,852	3,44%	1,509	0,594	13,37%
**Q53SF7**	Cordon-bleu protein-like 1 OS=Homo sapiens GN=COBLL1 PE=1 SV=2	0,897	-0,157	97,28%	1,758	0,814	0,50%
**Q58FF6**	Putative heat shock protein HSP 90-beta 4 OS=Homo sapiens GN=HSP90AB4P PE=5 SV=1	4,042	2,015	0,00%	1,040	0,057	100,69%
**Q5RKV6**	Exosome complex component MTR3 OS=Homo sapiens GN=EXOSC6 PE=1 SV=1	1,330	0,412	76,64%	1,769	0,823	0,43%
**Q8N5K1**	CDGSH iron-sulfur domain-containing protein 2 OS=Homo sapiens GN=CISD2 PE=1 SV=1	0,567	-0,818	1,36%	0,636	-0,652	0,75%
**Q8NEZ2**	Vacuolar protein sorting-associated protein 37A OS=Homo sapiens GN=VPS37A PE=1 SV=1	1,265	0,340	89,30%	0,485	-1,044	0,00%
**Q8WVY7**	Ubiquitin-like domain-containing CTD phosphatase 1 OS=Homo sapiens GN=UBLCP1 PE=1 SV=2	1,930	0,949	1,14%	0,969	-0,046	96,18%
**Q93070**	Ecto-ADP-ribosyltransferase 4 OS=Homo sapiens GN=ART4 PE=2 SV=2	2,120	1,084	0,18%	1,746	0,804	0,59%
**Q96GW9**	Methionine--tRNA ligase, mitochondrial OS=Homo sapiens GN=MARS2 PE=1 SV=2	1,139	0,188	97,48%	1,951	0,964	0,03%
**Q96HE7**	ERO1-like protein alpha OS=Homo sapiens GN=ERO1L PE=1 SV=2	0,901	-0,151	96,74%	0,651	-0,618	1,41%
**Q99622**	Protein C10 OS=Homo sapiens GN=C12orf57 PE=1 SV=1	0,317	-1,658	0,00%	0,586	-0,771	0,09%
**Q99988**	Growth/differentiation factor 15 OS=Homo sapiens GN=GDF15 PE=1 SV=3	1,862	0,897	2,03%	1,325	0,406	63,48%
**Q9BQE5**	Apolipoprotein L2 OS=Homo sapiens GN=APOL2 PE=1 SV=1				0,688	-0,539	4,83%
**Q9BU89**	Deoxyhypusine hydroxylase OS=Homo sapiens GN=DOHH PE=1 SV=1	1,039	0,055	99,94%	1,888	0,917	0,07%
**Q9GZM5**	Protein YIPF3 OS=Homo sapiens GN=YIPF3 PE=1 SV=1	1,795	0,844	3,78%	0,854	-0,227	75,18%
**Q9H299**	SH3 domain-binding glutamic acid-rich-like protein 3 OS=Homo sapiens GN=SH3BGRL3 PE=1 SV=1	0,550	-0,862	0,74%	0,454	-1,139	0,00%
**Q9H3K6**	BolA-like protein 2 OS=Homo sapiens GN=BOLA2 PE=1 SV=1	0,891	-0,166	94,93%	0,657	-0,606	1,69%
**Q9H4I2**	Zinc fingers and homeoboxes protein 3 OS=Homo sapiens GN=ZHX3 PE=1 SV=3	0,900	-0,151	96,95%	0,657	-0,605	1,66%
**Q9NP97**	Dynein light chain roadblock-type 1 OS=Homo sapiens GN=DYNLRB1 PE=1 SV=3	0,676	-0,564	21,13%	0,637	-0,650	0,77%
**Q9NZT2**	Opioid growth factor receptor OS=Homo sapiens GN=OGFR PE=1 SV=3	0,553	-0,855	0,79%	0,731	-0,451	14,41%
**Q9NZZ3**	Charged multivesicular body protein 5 OS=Homo sapiens GN=CHMP5 PE=1 SV=1	0,978	-0,033	99,42%	0,671	-0,576	2,61%
**Q9P2D3**	HEAT repeat-containing protein 5B OS=Homo sapiens GN=HEATR5B PE=1 SV=2	0,562	-0,831	1,12%	0,986	-0,020	98,15%
**Q9UIG0**	Tyrosine-protein kinase BAZ1B OS=Homo sapiens GN=BAZ1B PE=1 SV=2	1,275	0,351	87,99%	1,752	0,809	0,54%
**Q9UII2**	ATPase inhibitor, mitochondrial OS=Homo sapiens GN=ATPIF1 PE=1 SV=1	0,756	-0,404	58,80%	0,565	-0,823	0,03%
**Q9UK22**	F-box only protein 2 OS=Homo sapiens GN=FBXO2 PE=1 SV=2	0,568	-0,816	1,33%	0,780	-0,358	37,06%

**Q9UNL2**	Translocon-associated protein subunit gamma OS=Homo sapiens GN=SSR3 PE=1 SV=1	**0,600**	**-0,737**	**3,36%**	0,966	-0,050	95,56%
**Q9Y2K7**	Lysine-specific demethylase 2A OS=Homo sapiens GN=KDM2A PE=1 SV=3	0,964	-0,053	99,25%	**1,686**	**0,753**	**1,41%**
**Q9Y6K5**	2~-5~-oligoadenylate synthase 3 OS=Homo sapiens GN=OAS3 PE=1 SV=3	**0,429**	**-1,220**	**0,00%**	0,769	-0,380	31,08%

Down-expressed
Up-expressed

Significant (FDR<1%)
Significant (1%<FDR<5%)
Non-significant FDR>5%

2.10. Table S1. Summary of quantitated proteomics analyses from MCF7-Tet-On-SrcDN mammospheres.

Table S2. Detailed information for antibodies used in this work.

Name	Type	Supplier	Catalog #	Origin	Dilution Ratio WB*-FC/IF**-IHC***
β-Actin	mouse monoclonal	Sigma-Aldrich (Merck)	A5441	San Luis, MI, USA	1:1000-….-….
ALDH1	mouse monoclonal	BD-Biosciences	#611194	Franklin Lakes, NJ, USA	1:500-….-….
Cadherin-E	mouse monoclonal	A. Cano		IIB (UAM/CSIC)	1:200_1:50_1:20
Cadherin-P (clone 56C1)	mouse monoclonal	Novocastra Laboratories	#610227	Newcastle, UK	….-….-1:200
CD44-APC	mouse monoclonal	BD-Biosciences	#559942FIT	Franklin Lakes, NJ, USA	….-1:20-….
CD24-PE	mouse monoclonal	BD-Biosciences	#555428	Franklin Lakes, NJ, USA	….-1:20-….
Catalase	mouse monoclonal	Sigma-Aldrich (Merck)	#C0979	San Luis, MI, USA	1:1000_…._….
Cyclin D1 (H-295)	rabbit polyclonal	Santa Cruz Biotechnology, Inc.	sc-753	Dallas, TX, USA	1:1000-….-….
c-Src (MAb-327)	mouse monoclonal	J.S. Brugge (Harvard University)		Harvard University, MA, USA	1:1000-….-….
c-Src (MAb EC10)	mouse monoclonal	Millipore (Merk)	#05-185	Billerica, MA, USA	1:1000-….-….
c-Src-pY418	rabbit polyclonal	Invitrogen	#44660G	Camarillo, CA, USA	1:1000-….-….
Envision-peroxidase anti-mouse polymer labeled with HRP	goat polyclonal	Dako Corporation	K4000	Carpinteria, CA, USA	…._….._1:100
ESA-FITC	mouse monoclonal	Biomeda Corp.	FM010	Foster City, CA, USA	…._…._1:20
Fak	rabbit polyclonal	Santa Cruz Biotechnology, Inc.	Sc-557	Dallas, TX, USA	1:1000-….-….
Fak-pY397	mouse monoclonal	BD-Biosciences	#611722	Franklin Lakes, NJ, USA	1:1000-….-….
GAPDH (clone 6C5)	mouse monoclonal	Millipore (Merk)	CB1001	Billerica, MA, USA	1:4000_…._….
Glut-1	rabbit polyclonal	Santa Cruz Biotechnology, Inc.	Sc-7903	Dallas, TX, USA	1:1000-….-….
HK2	rabbit polyclonal	Cell Signaling Technology	#C64G5	Danvers, MA, USA	1:1000-….-….
LDHA	rabbit polyclonal	Cell Signaling Technology	#3582	Danvers, MA, USA	1:1000-….-….
LDHA-pY10	rabbit polyclonal	cell Signaling Technology	#8176	Danvers, MA, USA	1:1000-….-….
Mn-SOD	rabbit polyclonal	Millipore (Merck)	#06-984	Billerica, MA, USA	1:1000-…._….
MCT-1	mouse monoclonal	Santa Cruz Biotechnology, Inc.	Sc-365501	Dallas, TX, USA	1:1000-….-….
Nanog	rabbit polyclonal	Millipore (Merk)	#AB9220	Billerica, MA, USA	1:1000-….-….
Oct3/4	goat polyclonal	Santa Cruz Biotechnology, Inc.	Sc-8629	Dallas, TX, USA	1:100-….-….
PARP (clone C-2-10)	mouse monoclonal	Biomol Gmbh	SA-249	Hamburg, Gemany	1:1000-….-….
α-Tubulin	mouse monoclonal	Sigma-Aldrich (Merck)	T9026	San Luis, MI, USA	1:8000-….-….

Western Blotting (WB)*
Flow-cytometry (FC)/Immunofluorescence (IF)**
Immunohistochemistry (IHC)***

2.11. Table S2. Antibodies employed in these studies.

1

HUMAN CELL LINE STR PROFILE REPORT

CELL LINE DESCRIPTION according to customer: MCF7 (ATCC ® HTB-22/CVCL_0031)

Sample Submitted By:

Jorge Martín Pérez
Instituto de Investigaciones Biomédicas. CSIC
jmartin@iib.uam.es

Date: 13.12.2018

Microsatellite Run Number (pdf attached): M192

Laboratory:

Genomics Core Facility.
Instituto de Investigaciones Biomédicas "Alberto Sols" CSIC-UAM.
28029 Madrid
00 34 91 585 44 74 (Tel.) 00 34 91 585 44 01 (Fax)
genomica@iib.uam.es

STR PROFILE DATA:

STR PROFILE OBTAINED		EXPECTED
D5S818	12	11,12
D13S317	11	11
D7S820	8,9	8,9
D16S539	11,12	11,12
vWA	14,15	14,15
TH01	6	6
TPOX	9,12	9,12
CSF1PO	10	10
D21S11	30	
Amelogenin	X	X
RESULTS		
Number of shared alleles: 15		
Number of alleles in database: 16		
Percent match: 93%		

TEST RESULTS:
Query profiles were sent against ATCC database
Cell lines with ≥80% match are considered to be related (i.e common ancestor).
Cell lines with 55%-80% match require further profiling

- ✓ The submitted sample profile is human and matches 93% with customer cell line description
- The submitted sample profile is human, but does not match customer cell line description
- No human STRs are amplified

DATABASE OUTPUT:

%Match	ATCC® Number	Designation	D5S818	D13S317	D7S820	D16S539	vWA	TH01	AMEL	TPOX	CSF1PO
93.0	HTB-22	MCF7Breast AdenocarcinomaHuman	11,12	11	8,9	11,12	14,15	6	X	9,12	10

METHOD:

STR ANALYSIS	
STR amplification kit	GenePrint^{R} 10 System (Promega)
STR profile analysis software	GeneMapper® v3.7 (Life Technologies)
Genomic Analyzer System	ABI 3130 XL (Applied Biosystems)
DNA source	Cultured cells; cultured cells pellet
DNA isolation method	DNeasy blood and tissue kit (Qiagen)
DNA quantification method	Qubit 2.0 Fluorometer (Life Technologies)
Amount of DNA/amplification	4 ng

The *GenePrint*® 10 System allows co-amplification and three-color detection of ten human loci: TH01, TPOX, vWA, Amelogenin, CSF1PO, D16S539, D7S820, D13S317, D21S11 and D5S818. These loci collectively provide a genetic profile with a random match probability of 1 in 2.92 × 10^9 and are used for human cell line and tissue authentication and identification and human cell line cross-contamination determination. Samples are processed using the ABI Prism® 31300xl Genetic Analyzer. Data are analyzed using GeneMapper® ID-X v1.2 software (Applied Biosystems). STRs profiles are sent for comparison against cell line date bases like ATCC (American Type Culture Collection), DSMZ (Deutsche Sammlung von Mikrorganismen and Zellkulturen)…

2

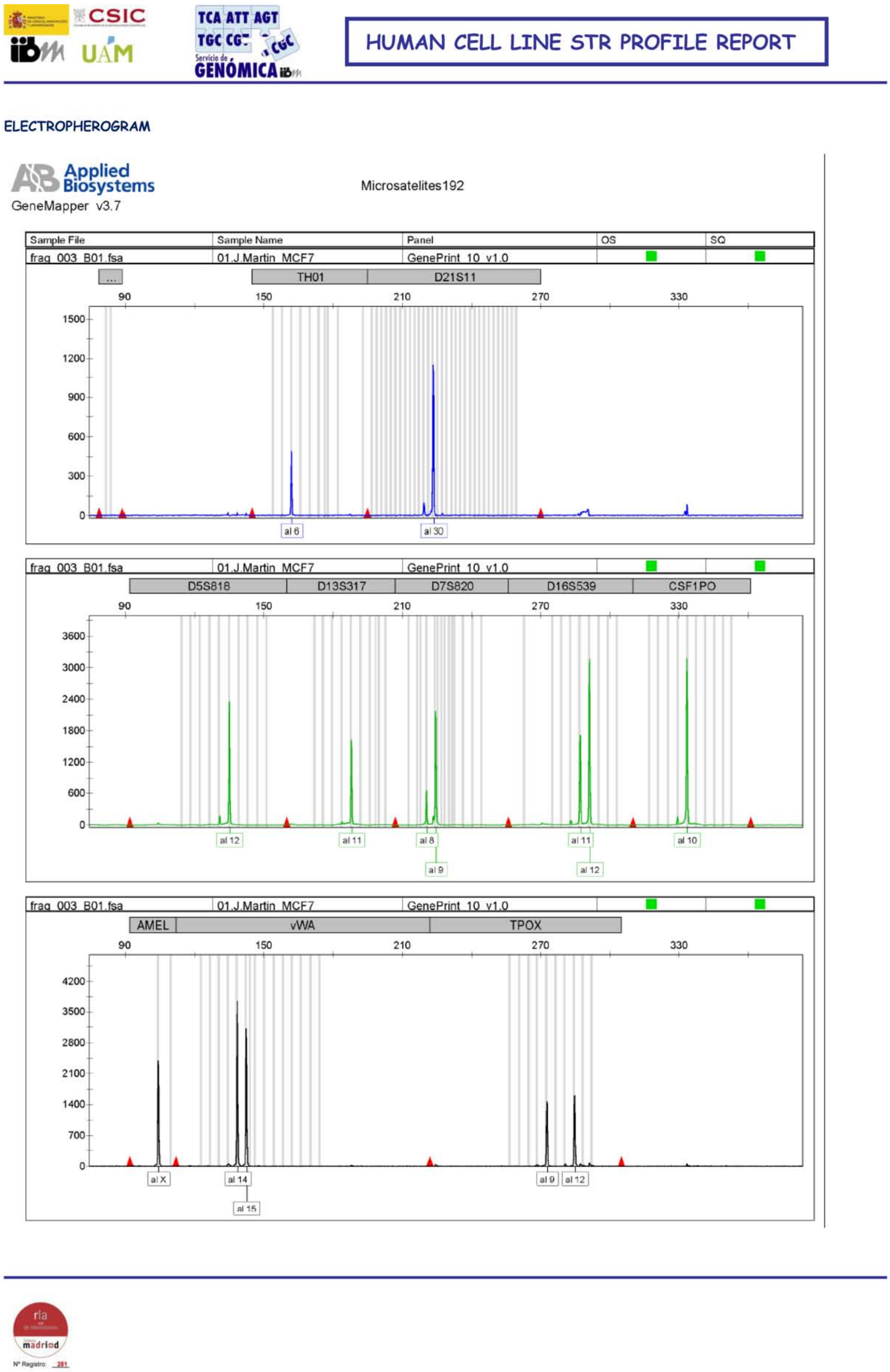

2.12. Reports of MCF7 cell line authentication. MCF7 cell line was authenticated by short-tandem-repeat analysis (GenePrintR 10 System from Promega, and GeneMapper v3.7 STR profile analysis software, Life Technologies).

Uncropped Immunoblots from Figure_1

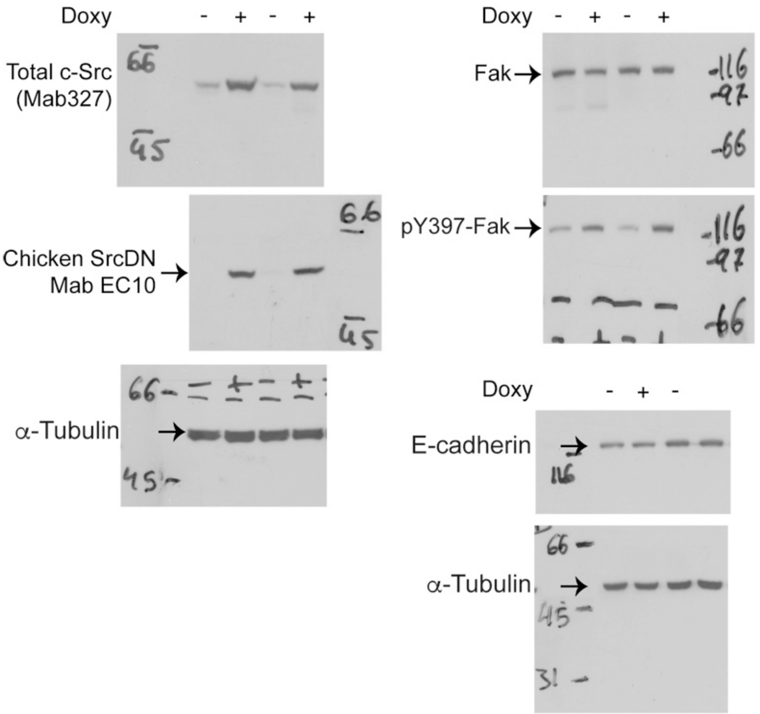

Uncropped Immunoblots from Figure_2

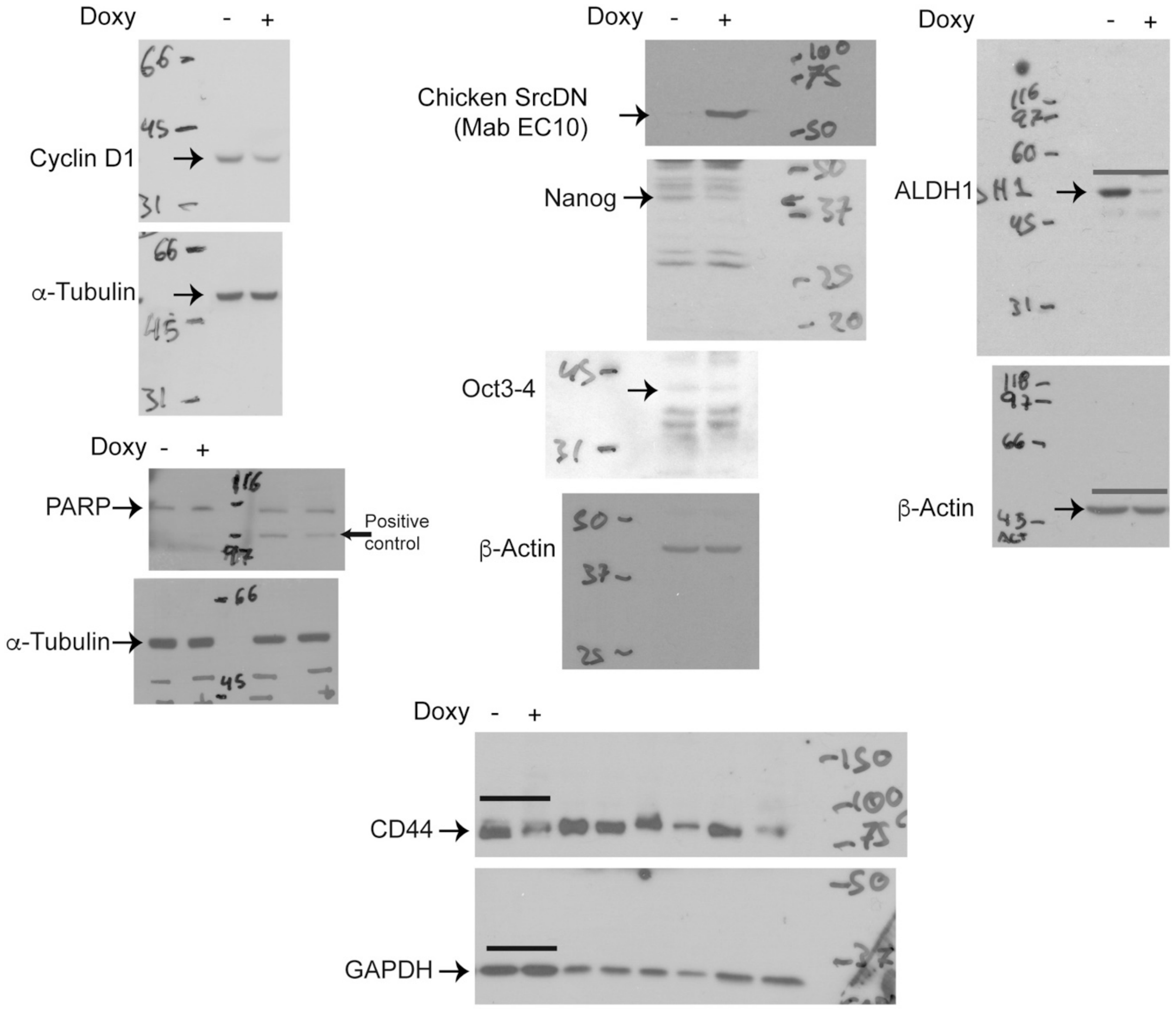

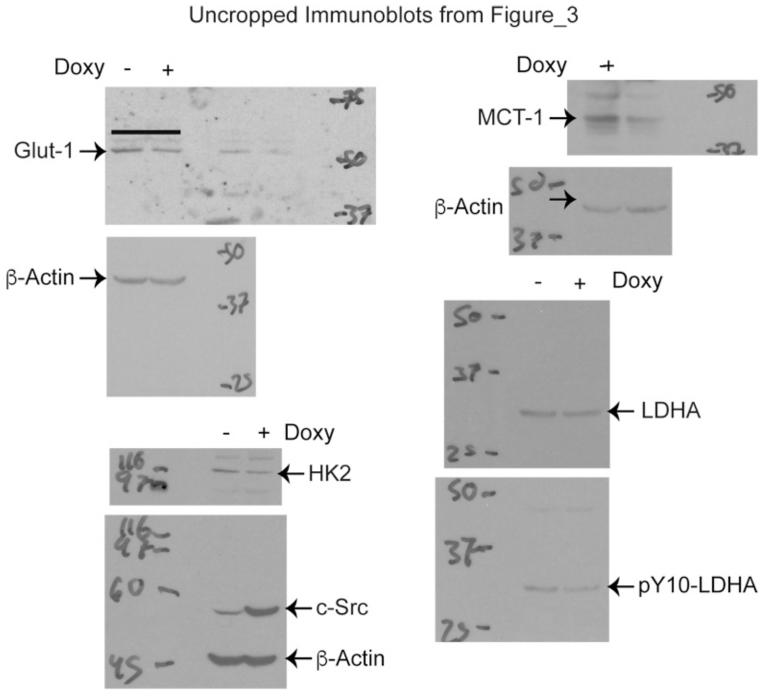

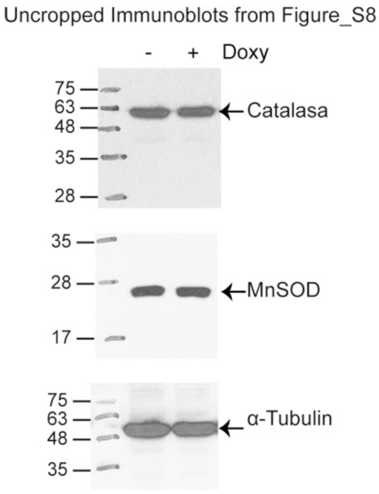

2.13. Figure. Uncropped scan of the western blots of the manuscript. Since it was difficult (time consuming) to obtain large amounts of samples, we cut the transfer membranes to incubate each of them with antibodies to obtain different results from a single immunoblotting membrane.

MCF7-Tet-On-SrcDN_Mammospheres (FIGURE 1)

CD24-neg		CD24-pos		
Control	Doxy	Control	Doxy	
9199	42294	6915	21677	Total c-Src

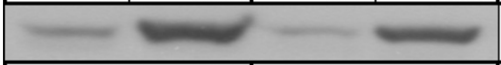

31423	33411	30422	30583	α-Tubulin

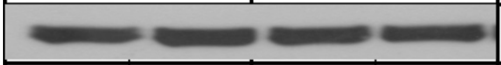

**0,29**	**1,27**	**0,23**	**0,71**	Total c-Src/α-Tubulin
1,00	4,32	1,00	3,12	

CD24-neg		CD24-pos		
Control	Doxy	Control	Doxy	
13667	20221	9692	20144	pY397-Fak

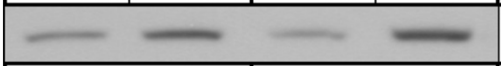

30638	24678	24428	26256	Fak

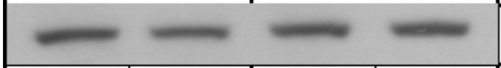

**0,45**	**0,82**	**0,40**	**0,77**	pY397-Fak/Fak
1,00	1,84	1,00	1,93	

CD24-neg		CD24-pos		
Control	Doxy	Control	Doxy	
21413	21098	32962	31742	E-cadherin

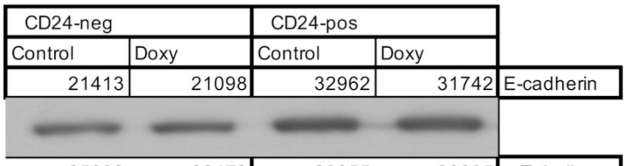

25399	22473	28955	29835	α-Tubulin

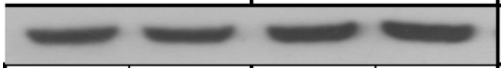

**0,84**	**0,94**	**1,14**	**1,06**	E-cadherin/α-Tubulin
1,00	1,11	1,00	0,93	

18

MCF7-Tet-On-SrcDN Mammospheres (FIGURE 2)

Control	Doxy	
45267	26350	cyclin D1

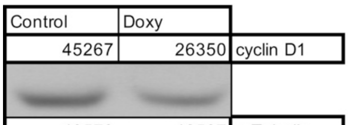

43579	42597	α-Tubulin

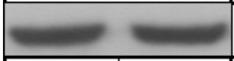

**1,04**	**0,62**	cyclin D1/α-Tubulin
1,00	0,60	

Control	Doxy	
21042	2790	ALDH1

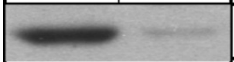

16771	12098	β-Actin

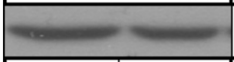

**1,25**	**0,23**	ALDH1/β-Actin
1,00	0,18	

Control	Doxy	
37282	17808	CD44

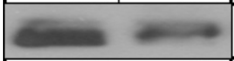

35727	38638	GAPDH

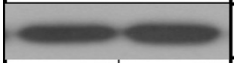

**1,04**	**0,46**	CD44/GAPDH
1,00	0,44	

Control	Doxy	
40750	26426	Nanog

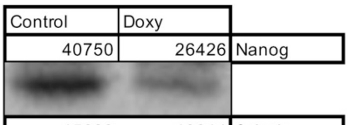

15623	18214	β-Actin

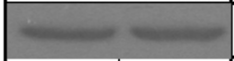

**2,61**	**1,45**	Nanog/β-Actin
1,00	0,56	

Control	Doxy	
28840	18688	Oct3-4

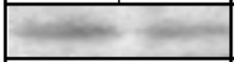

15623	18214	β-Actin

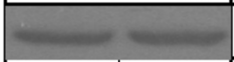

**1,85**	**1,03**	Oct3-4/β-Actin
1,00	0,56	

19

MCF7-Tet-On-SrcDN_Mammospheres (FIGURE 3)

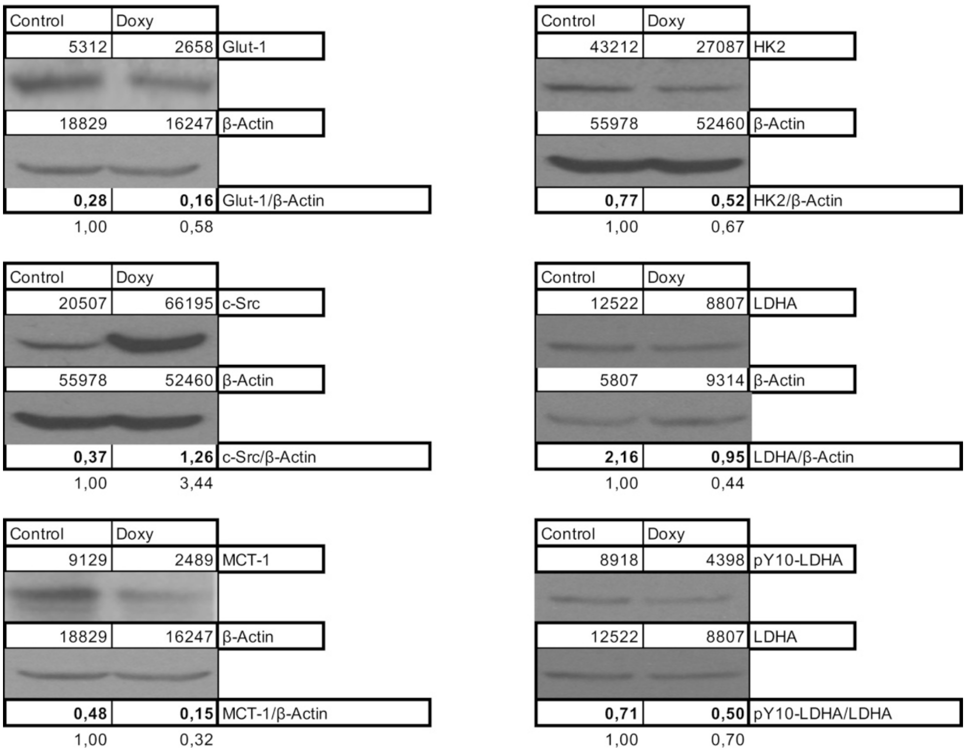

MCF7-Tet-On-SrcDN Mammospheres (FIGURE S8)

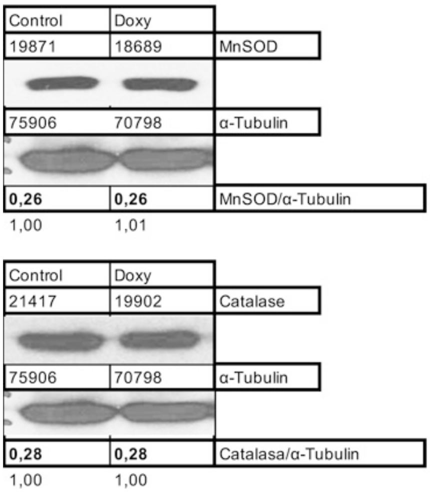

2.13. Quantification of immunoblots.

3. References

1. Cruz-Bermudez, A.; Vallejo, C.G.; Vicente-Blanco, R.J.; Gallardo, M.E.; Fernandez-Moreno, M.A.; Quintanilla, M.; Garesse, R. Enhanced tumorigenicity by mitochondrial DNA mild mutations. *Oncotarget* **2015**, *6*, 13628-13643, doi:10.18632/oncotarget.3698.
