# Supplementary material for: c-Src functionality controls self-renewal and glucose metabolism in MCF7 breast cancer stem cells
Source: PLoS One. 2020 Jul 16;15(7):e0235850. doi: 10.1371/journal.pone.0235850 (PMC7365443; doi:10.1371/journal.pone.0235850)
Supplement: Attachment — Submitted filename: Reviewers_Repply_03-06-2020.pdf [file pone.0235850.s002.pdf]

Dear Editor and Reviewers, to be able to see the proteomic deposit please see:

Project Name: c-Src functionality controls self-renewal, tumorigenicity and glucose metabolism in breast cancer stem cells
Project accession: PXD017789
Project DOI: 10.6019/PXD017789

Reviewer account details:
Username: reviewer69051@ebi.ac.uk
Password: RX7tB4jU

Reviewer 1

Dear Reviewer, we very much appreciate your observation that allowed us to see several defects on our manuscript. The revised manuscript contains new experiments and supplementary figures. Hopefully, we have been able to solve these imperfections that should permit you to accept our manuscript.

Figure 1: *The CD44-high/CD24-low population of BCSCs was isolated from MCF7-Tet-On-SrcDN cultures without induction of SrcDN, and then cells were plated to form mammospheres. However,*

A. *it is not clear from the legend how long the mammospheres, once formed, were treated with DOX prior to the immunoblotting analysis or what happened to the acinar structures during the DOX treatment period, e.g., do they maintain their morphology.*

Mammospheres were treated for three passages with Doxy (2 μg/mL) in the culture media. No changes in the morphology were detected upon treatment with Doxy. Thus, we have changed the legend of Figure 1.C as follow:
"Immunoblots of total c-Src, SrcDN, p-Y397-Fak/Fak, and E-cadherin, using α-Tubulin expression as loading control from $CD24^-$ cells forming mammospheres and $CD24^+$ cells in absence (Control) or presence of Doxy (2 μg/mL) for induction of SrcDN during three passages. The ratios of the proteins Total c-Src, SrcDN, and E-cadherin with the loading control α-Tubulin, or pY397-Fak/Fak were calculated and referred to -Doxy (Control) considered as 1. These are representative results from 3 independent experiments."

B. *It is also not clear what happens to the phenotype of BCSC cells once a mammosphere has been formed - do all the cells retain their BCSC phenotype or have some of them differentiated back into CD44-high/CD24-high cells.*

Although in the mammosphere culture is probable that some cells differentiated back into CD44-high/CD24-high cells, the pluripotent marker analysis by immunoblotting showed that the mammosphere culture cells retain BCSC phenotype.

C. *The authors report that when mammospheres were dissociated into single cells and then re-plated they could form mammospheres, but information about the efficiency of this process was not provided. If the dissociated cells are re-analyzed by FACS, what fraction are CD44-high/CD24-low and what fraction are CD44-high/CD24-high.*

Only a very small portion of MCF7-Tet-On-SrcDN cells are able to form mammospheres, the vast majority of them die by anoikis in suspension because these cells need to attach to a surface to grow. Even so, it is very likely that a mammosphere is not only made up of CD44-hig/CD24-low cells. But over the course of several generations, cultures become enriched of CD44-high/CD24-low cells, as more mammospheres are formed after plating the same number of cells in each successive generation. All the experiments were carried out with cells from mammospheres in the 3rd generation based on the fact that at this generation the markers of pluripotency were detected, and considering that later generations can modify the cells as described by M. Iglesias et al {Manuel Iglesias, 2013 # 6012}, this being the best way to get enough cells for the assays.

To show the enrichment of the CD24- population forming mammospheres, we are including the evaluation of the three generations of mammospheres at the Supplementary S2 Fig.

Three generations of Mammospheres from MCF7-Tet-On-SrcDN

	1st generation		2nd generation		3rd generation	
	- Doxy	+ Doxy	- Doxy	+ Doxy	- Doxy	+ Doxy
Exp. 1	7.76	2.84	9.99	5.16	10.40	6.70
Exp. 2	4.99	3.44	7.95	5.43	10.80	8.80
Exp. 3	5.73	3.73	6.50	4.20	10.30	8.10
Exp. 4	8.56	4.87	8.25	6.44	9.60	3.50
Exp. 5	6.60	4.00	10.50	7.00	8.20	3.90
Mean	6.73	3.78	8.64	5.65	9.86	6.21
SD	1.5	1.0	1.6	1.0	1.0	2.0
t-Student	0.004		0.009		0.014	
	p<0.01 **		p<0.01 **		p<0.5 *	

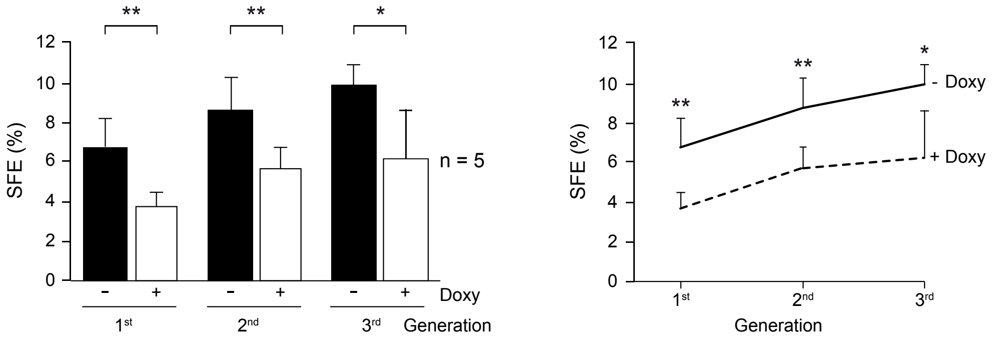

Furthermore, as we previously shown in MCF7-Tet-On-SrcDN, SrcDN significantly reduced tumorigenesis, causing regression on established tumors in nude mice [Ref. 23 in initial manuscript]. However, it was unclear whether SrcDN affected functionality of $CD24^-$ subpopulation. Thus, we carried out sometime ago a pilot test for $CD24^-$ and $CD24^+$ cells tumorigenicity by subcutaneously injecting $1x10^4$ $CD24^-$ cells (cells derived from mammospheres) in the left flank of nine female nude mice, and $5x10^5$ $CD24^+$ cells in the right flank, in a ratio 1/50 of $CD24^-/CD24^+$, similar to cell proportion isolation by FACS. $CD24^-$ cells were tumorigenic, producing 8/9 tumors (89%), while $CD24^+$ cells showed residual tumorigenicity, 1/9 tumors (11%), which was consistent with published data [Ref. 3, 4, 24 in initial manuscript]. CD24-mammosphere cells are tumorigenic and $CD24^+$ are not tumorigenic. In parallel, groups of nine animals received Doxy (2 mg/ml) in the drinking water, which induced SrcDN expression in injected cells. Under this condition, tumorigenicity of $CD24^-$ cells was reduced by 67%, only 2 tumors were generated (22%), while the group of $CD24^+$ cells remained unchanged, 1/9 tumors at the right flank of the mice. This pilot experiment, indicated that the cells contained in mammospheres were mainly $CD24^-$ and tumorigenic, additionally they also suggested that Src functionality is relevant for tumorigenesis of $CD24^-$-MCF7-derived cells. Since we are unable to carry out a dose/response tumorigenic study, we have not included these data in the manuscript.

Nevertheless, following Reviewer' suggestion, we have dissociated mammospheres at the 3rd generation, labelled cells with isotypic immunoglobulins as control, as well as with anti-CD24-PE and CD44 -APC and analyzed them by flow cytometry. The results, collected now as Figure S3 (please, see below) shows that in mammospheres Control (untreated with Doxy) most of cells are $CD24^-/CD44^+$. In contrast, in mammospheres treated with Doxy (2 μg/mL), a large number of cells are $CD24^+/CD44^+$. In addition, the analysis of the expression level of these markers (MFC, mean

fluorescence channel) indicates that, along with an increase of expression of CD24, treated mammospheres show a reduced expression of CD44. These results agree with those presented in Fig 2, displaying that SFE, as well as expression of CD44, ALDH1, Nanog and Oct3-4, were also reduced in mammospheres expressing SrcDN upon Doxy (2 μg /mL) treatment.

A

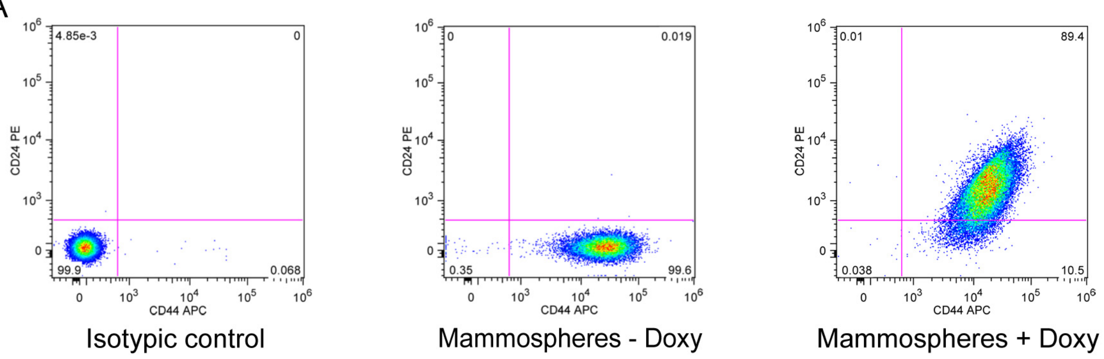

B

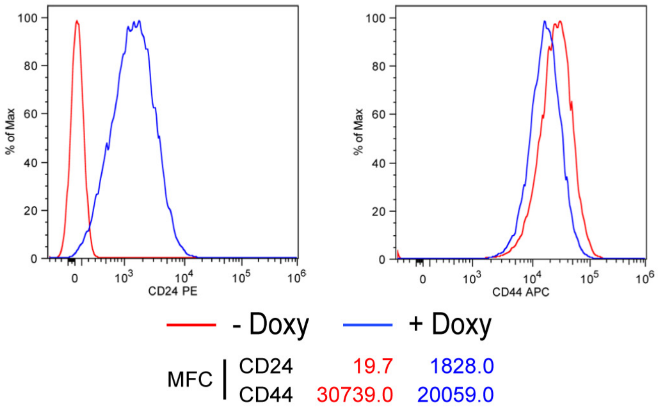

D. *If the cells are treated with DOX either prior to (i.e. as mammospheres) or during the re-plating process with DOX, how does this affect the efficiency of mammosphere formation (see point 2)?*

Cells were initially plated in 6-well ultralow attachment plates at $2x10^3$ cells/well in mammosphere culture media without Doxy. Fifteen days later, mammospheres were dissociated into single cells that were plated in 6-well ultralow attachment plates at $2x10^3$ cells/well in mammosphere culture media, 3 wells without Doxy (Control) and 3 wells with Doxy (2 μg/ml). These two groups were maintained during the three generations, as well as during the re-plating.

2. *Figure 2A:* *It is not clear from the legend or text exactly what was done in this experiment. Were the single cells that were plated obtained from mammospheres that had been pretreated with DOX, or were the cells continuously treated with DOX during plating and mammosphere outgrowth? This needs to be spelled out in the legend.*

Dear Reviewer you are absolutely right, it was so obvious for us that we have forgotten to describe essential details of our experimental work. Thank very much for reminding us that we need to define some important parameters of our experiments.

The adherent MCF7-Tet-On-SrcDN cells were not pretreated with Doxy before they were plated in mammosphere culture conditions. We have included this information in the revised version of the Manuscript at the Material and method in the Sphere Formation Efficiency (SFE) protocol (page 6, line 135):

Briefly, single cell suspensions from adherent MCF7-Tet-On-SrcDN cultures were plated in 6-well ultralow attachment plates (Falcon, Corning Life Science) at $2x10^3$ cells/well in mammosphere culture media [serum-free DMEM/Han's F12 media (1:1), BSA (4 mg/mL), EGF (20 ng/mL) and bFGF (20 ng/mL), insulin (5 µg/mL), hydrocortisone (5 µg/mL)], to obtain mammospheres. Fifteen days later, mammospheres were dissociated into single cells that were plated in 6-well ultralow attachment plates at $2x10^3$ cells/well in mammosphere culture media, 3 wells without Doxy (Control) and 3 wells with Doxy (2 µg/mL). During the experiments Doxy was renewed every 3 days in cultures. Each mammosphere generation was cultured about 15 days. Mammospheres (sphere-like structures with diameter ≥ 50 µm) were clearly detected by optical phase contrast microscope (Nikon-Eclipse TS100, 4x magnification). For mammosphere dissociation, they were collected in a sterile tube and allow them to sediment, the media was removed with a pipette and trypsinized in Tris-Saline pipetting up and down to facilitate mammosphere dissociation, cells were then collected by centrifugation to remove trypsin, washed with mammosphere media and counted. Single cells were seeded again for mammosphere formation in 6-well ultralow attachment plates at $2x10^3$ cells/well. The experiment ended at third generation of mammosphere formation. SFE at the third generation was then calculated as number of spheres formed per number of seeded cells and expressed as % means ± SD. The SFE experiments were repeated 5 times, each one of them was carried out in triplicates (p<0.05*).

Also, at the legend of figure 2A, we have included that:

(**A**) The Sphere Formation Efficiency (SFE) was determined in MCF7-Tet-On-SrcDN. Thus, single cell suspensions from adherent cells were plated in 6-well ultralow attachment plates at $2x10^3$ cells/well and cultured in mammosphere medium. Fifteen days later, mammospheres were dissociated into single cells that were divided into two groups: Control (-Doxy) and Doxy treated (2 µg/mL). Doxy-treatment was maintained for three generations, and renewed every 3 days. SFE at third generation was calculated as number of spheres formed per number of seeded cells and expressed as % means ± SD. The SFE experiments were repeated 5 times, each one of them was carried out in triplicates. (p<0.05*).

As shown above, we included in the Revised Manuscript as NEW Supplementary Fig 2 (S2 Fig) the excel of the counts of mammospheres from Control and Doxy (2µg/mL) of the five independent experiments (carried out in triplicates) and the mean value -/+ standard deviation and the p value of three mammospheres generations, which showed an enrichment in mammospheres from the first to the third generation.

3. ***Figure 2B:*** *How long was SrcDN induced for in the mammospheres prior to harvesting for the proteomic analyses?*

We should point out that Figure 2B is not referring to proteomic analyses. Probably, the Reviewer was referring to the immunoblotting in the proteomic analyses.

Nevertheless, following the Reviewer 1 comments, the legend of Fig 2 A, B in the Revised Manuscript will be as follow:

FIGURE 2. Self-renewal of mammospheres derived from MCF7.
(**A**) The Sphere Formation Efficiency (SFE) was determined in MCF7-Tet-On-SrcDN. Thus, single cell suspensions from adherent cells were plated in 6-well ultralow attachment plates at $2x10^3$ cells/well and cultured in mammosphere medium. Fifteen days later, mammospheres were dissociated into single cells that were divided into two groups: Control (-Doxy) and Doxy treated (2 µg/mL). Doxy-treatment was maintained for three generations, and renewed every 3 days. SFE at third generation was calculated as number of spheres formed per number of seeded cells and expressed as % means ± SD. The SFE experiments were repeated 5 times, each one of them was carried out in triplicates. (p<0.05*). (**B**) Total cell extracts from the 3rd generation of mammospheres treated as above (panel A) were used to determine by immunoblotting expression of cyclin D1, PARP, with α-Tubulin, as a loading control, and SrcDN, Nanog, Oct3/4, ALDH1, and CD44, employing GAPDH or β-Actin, as a loading control. The net quantification of the gel bands after subtracting the background was carried out with ImageJ program and expressed in arbitrary units. These are representative results from 3 independent experiments. The ratios of the proteins SrcDN, Nanog, Oct3/4, ALDH1, and CD44 and their loading controls

GAPDH or β-Actin, or pY10-LDHA/LDHA were calculated and referred to -Doxy (Control) considered as 1.

Addressing the Reviewer question: "*How long was SrcDN induced for in the mammospheres prior to harvesting for the proteomic analyses?*"

The quantitative proteomic analyses were carried out with Control or Doxy treated (2 μg/mL) third generation mammospheres. We have included in the revised version of the Manuscript at the Material and method in Quantitative proteomic analyses protocol that: MCF7-Tet-On-SrcDN derived mammospheres at 3rd generation from two independent experiments cultured either in the absence (Control) or presence of Doxy (2 μg/mL). ….

4. *Table 1:* *It is unclear from the text and the supplementary materials how many total proteins were identified through the MS analysis, i.e. the 15 protein decreases and 4 protein increases need to have a denominator of the total number of proteins identified. By comparative immunoblotting of control and DOX-induced MCF7-SrcDN mammospheres, the authors showed that the levels of several proteins involved in stem cell function and the glycolytic pathway were decreased upon SrcDN induction. However, it is not clear how many of these proteins were also found to be downregulated in their proteomic dataset - none of the proteins tested by immunoblotting is listed in Table S1, but I did not check the data deposited in the ProteomeXchange.*

The denominator of the total number of proteins identified with at least two peptides were 2,759 proteins. From those, only 101 proteins were differentially expressed with an FDR < 5%. We will introduce these data in the result section: Result/Proteomic analyses of mammospheres (page 12, line 286) the following description:
Proteomic analyses identified 2,759 proteins with at least two peptides. From those, only 101 proteins were differentially expressed with an FDR < 5% (S1 Table). In the Table I only proteins having at least two quantitated peptides and <5% quantitation FDR with the same tendency in both experiments were included. Thus, induction of SrcDN by Doxy (2 μg/mL) diminished the levels of fifteen proteins, and increased the levels of four of them (Table I, and S4 Fig). The most increased protein in Doxy/Control is c-Src, which most likely should correspond to SrcDN induced by Doxy-treated mammospheres, as the identity of *Homo Sapiens* and *Gallus-Gallus* c-Src at the amino acid sequences is 94%. Results obtained were submitted to GSEA and found that glycolysis was significantly reduced in MCF7-mammospheres upon SrcDN induction (S5 Fig), which was consistent with data from Table I.

Thus, we have changed the legend of the supplementary S1 Table as:

"2.6. Table S1. Summary of differentially expressed proteins obtained from proteomics analysis of MCF7-Tet-On-SrcDN mammospheres.".

As described in Material and methods, at Quantitative proteomic analysis: Only proteins having at least two quantitated peptides were considered in the quantitation. A 5% quantitation FDR threshold was estimated to consider the significant differentially expressed proteins.

TABLE I. Quantitative proteomic analysis of mammospheres derived from MCF7-Tet-On-SrcDN. The analysis was carried out in two independent experiments from the total cell extract of the 3rd generation mammospheres either untreated (Control) or Doxy-treated. Only proteins having at least two quantitated peptides and <5% quantitation FDR in both experiments are included in the table.

5. *Figure 2:* *Have the authors carried out a parallel RNA-seq analysis on control and DOX-induced MCF7-SrcDN cells to determine which of the observed changes in protein levels can be attributed to decreased levels of the cognate mRNAs (such an analysis does not seem to be*

reported in their prior studies on MCF7-Tet-On-SrcDN cells). It would be particularly interesting to know whether SrcDN expression affects the levels of Myc and HIF1 RNA expression, since these transcription factors are known to promote expression of the HK2 and LDHA genes. Also, are there any changes in the levels of expression of RNAs encoding mitochondrial ETC genes? Likewise, analysis of the differences in RNA expression patterns between CD44-high/CD24-low BCSCs and the CD44-high/CD24-high cells would be informative.

As Reviewer indicated, Myc and HIF1 promote the expression of enzymes implicated in glycolysis regulation. For this reason, we analyzed the protein expression levels of Myc and HIF1 by immunoblotting in three independent experiments. Expression of Myc was unaltered in mammospheres expressing SrcDN. Consequently, we did not report this result in the manuscript. Unfortunately, we were unable to detect HIF1 protein expression in any of the three independent samples by immunoblotting in either Control or SrcDN-expressing mammosphere cells. As suggested by the Reviewer we carried out analyses of Myc and HIF1 RNA expression by SYBR Green qRT-PCR using β-Actin as endogenous gene. RNA expression is reported as relative value referred to -Doxy (Control) considered as 1. In the revised manuscript we have included in the Supplementary Material a section for Materials and methods, as well as a new supplementary S6 Fig.

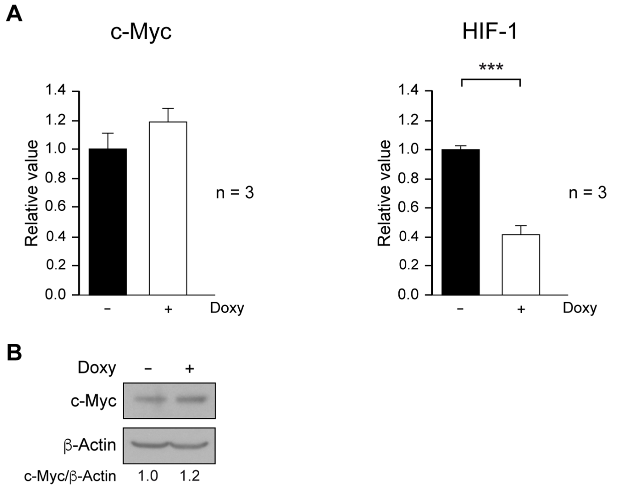

Supplementary Figure S6. c-Myc and HIF-1 expression in control and Doxy-treated (2 μg/mL) 3rd mammosphere generation. (**A**) Expression of c-Myc and HIF-1 by SYBR Green q-RT-PCR using β-actin as endogenous gene (see Materials and methods). Results are shown as Mean ± SD of relative levels in three independent experiments, considering arbitrarily -Doxy (Control) as 1 (***$p<0.001$). (**B**) Immunoblotting analysis of c-Myc expression employing β-actin as loading control. Results are referred to -Doxy (Control) considered as 1.

6. Figure 3: *Although an ~50% decrease in pY10 LDHA level upon DOX induction of SrcDN is reported in panel D, there is a concern about how accurate this value is - the low level of the pY10 immunoblot signal means that the percent decrease will be very dependent on how accurately the background signal, which needs to be subtracted, was estimated. A bigger decrease than the observed 50% in pY10 LDHA level might have been expected given the very high level of SrcDN expression compared to that of endogenous c-Src in the induced cells. In this regard, it has been reported (ref. 18) that the level of pY10 in MCF7 cells is very low compared to that in other more advanced breast cancer lines.*

Immunoblotting band was analyzed by ImageJ program and the value obtained from the background was always subtracted. Furthermore, for the pY10-LADH, the value of the immunoblotting band from ImageJ was normalized with value obtained from the ratio of LDHA/β-Actin bands

We know, as the Reviewer indicated us, that pY10-LDHA is very low in MCF7 compared with other less differentiated and more invasive breast cancer cell lines, such as the triple negative

MDA-MB-231 (Reference 18 in this manuscript). However, in our opinion it was important to analyze this aspect also in our model, the enriched BCSCs population derived from MCF7-Tet-On-SrcDN. Our result showed that the expression of SrcDN reduced the activity of LDHA and its phosphorylation level, suggesting that it could be responsible, at least in part, for the effects observed in this model.

Minor Points

1. *The first paragraph of the results is entitled "SrcDN reduced self-renewal of MCF7-BCSCs" but no such data are described in this section.*

Thank you very much, we changed the title in the Revised version of the Manuscript: "Isolation and characterization of enriched population of BCSCs from MCF7 cells"

2. *While many readers may know what MCF7 cells are, the authors should describe them in the Introduction, i.e. they are ER/PR-positive, HER2-negative, luminal A-type, non-metastatic human breast cancer cells.*

We thank the Reviewer for the indication, and we will introduce this definition in the Introduction of the Revised Manuscript

3. *Do the cellular phenotypes of DOX treated BCSCs revert when DOX is withdrawn, and if so how rapidly?*

While the total cell population of MCF7-Tet-On-SrcDN change its phenotype upon addition of Doxy inducing SrcDN [Refence 23 in the Manuscript, J Biol Chem. 2006;281(30):20851-64], we were unable to observe a morphological alteration upon induction of SrcDN by Doxy (2 μg/mL) in the mammosphere cultures. However, in a pilot experiment, by immunoblotting, we were able to detect reversion of SrcDN expression, as well as of ALDH1 and CD44 upon withdraw of Doxy, by washing mammospheres several times with media without Doxy and incubation of mammospheres for 96 h (data not shown). We have not carried out a time/course experiment for reversion.

4. *Page 12, second paragraph: The authors say that the endogenous c-Src cannot be distinguished from the exogenously expressed SrcDN by proteomic analysis, but this is not actually true, since they expressed chicken c-SrcDN in human cells, and there are a significant number of sequence differences between the two c-Src's, which would mean that the chicken and human c-Src proteins would generate unique peptides that could be quantified.*

We are expressing a chicken c-Src mutant (SrcDN), while the mammospheres were derived from human MCF7 cells endogenously expressing human c-Src wild type. BLASTp analysis of protein sequences identity between *Homo Sapiens* (Protein Accession Number: P12931.3) and *Gallus-Gallus* (Protein Accession: P00523.4) c-Src resulted in 94% identity at the amino acid sequences. Thus, all identified peptides at the quantitative proteomic analyses made here correspond to the common sequence of both *Homo Sapiens* and *Gallus-Gallus* c-Src. In addition, three different antibodies made for c-Src react with several species, including *Homo Sapiens* and *Gallus-Gallus*: 1. Mab 327 made long time ago in the laboratory of Johan S. Brugge (J. Virol. 48:352-60, 1983), which recognizes an antigen at the SH2 domain of c-Src; 2. Mab LA074 (it was produced by Quality Biothech, Camde, NJ, USA; Mol. Cell Biol. 12:1835-45, 1992; Mol. Endocrinol. 9:1461-67, 1995) recognizing the amino terminal portion of c-Src 8aa 2-17); 3. Src2, a polyclonal antibody that was produced by Santa Cruz Biotechnology made against the C-terminal fragment of c-Src, which also cross reacts with c-Src, Yes and Fyn, now replaced by sc-8056 Mab. All of these antibodies were used in the laboratory and react with human and chicken c-Src. It was because of these evidences that I did write that sentence. All said, even if I am not an expert in proteomics, I am persuaded that it could be possible to distinguish *Homo Sapiens* and *Gallus-Gallus* c-Src proteins with a very sophisticated and refined proteomic method. Indeed, the EC10 monoclonal antibody, used in this manuscript only recognizes chicken c-Src. Unfortunately, I have been long

searching for an antibody that exclusively reacts with human c-Src, and I had not been able to find it.

5. *Although technically more challenging because of the small cell numbers, it would have been interesting to analyze the sorted CD44-high/CD24-low BCSC MCF7 cell population directly for changes in protein levels, glycolysis rates, etc, compared to the CD44-high/CD24-high population.*

Unfortunately, we are presently unable to carry out these experiments.

Reviewer 2

Dear Reviewer, we very much appreciate your observation that allowed us to see several defects on our manuscript. The revised manuscript contains new experiments and supplementary figures. Hopefully, we have been able to solve these imperfections that should permit you to accept our manuscript.

Specific comments:

Figure 1:

1. Figure 1A, isotype control for setting up the gate for the FACS should be included.
2. Figure 1B, it is unclear the significance of this figure, since there is no comparison of spheres formed by CD24- and CD24+ groups. There is lack of scale bars.
3. Other methods have been used to isolate putative BCSCs, particularly using ALDH activity for the luminal breast cancers. To strengthen the claim on BCSCs, the authors should also consider using different ways to evaluate BCSCs.

1. Figure 1A, isotype control for setting up the gate for the FACS should be included.

We have included the isotype controls as Supplementary S1 Fig with the following figure legend: Supplementary Figure S1. Flow cytometric analysis of isotypic immunoglobulin labelling. Cells were incubated with FITC-mouse IgG1 (isotypic control for ESA staining, histogram on the left), with PE-mouse IgG2a (isotypic control for CD24 labelling) and APC-mouse IgG2b (isotypic control for CD44 labelling), dot plot on the right. The percentage of positive cells are indicated. Results from one representative experiment.

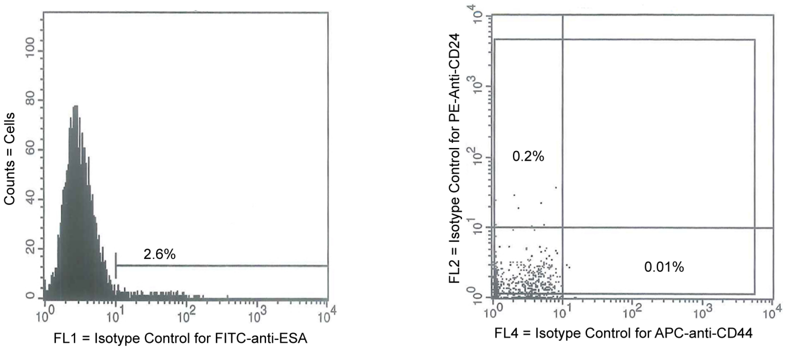

Supplementary Figure 1

2. Figure 1B, it is unclear the significance of this figure, since there is no comparison of spheres formed by CD24- and CD24+ groups. There is lack of scale bars.

In our experience and in the literature the CD24$^+$ cells (ESA$^+$-CD44$^+$-CD24$^+$) do not generate mammospheres. In Fig 1B, we wanted to show the morphology of mammospheres and the fact that these structures maintain the epithelial phenotype, as they express E-cadherin but not P-cadherin, which is a marker for mesenchymal cells of less differentiated and more aggressive breast tumors. We have included scale bars in the new Fig 1B.

3. Other methods have been used to isolate putative BCSCs, particularly using ALDH activity for the luminal breast cancers. To strengthen the claim on BCSCs, the authors should also consider using different ways to evaluate BCSCs.

As you indicate, Ginestier et al. published (Cell Stem Cell **2007**, 1:555-67, Reference 34 in our Manuscript) that ALDH1 defined a population enriched in BCSCs. Thus, as observed in Figure 2B, in addition of Nanog, Oct3/4, and CD44, we also determined by immunoblotting the expression of ALDH1, and found that it was clearly detected by immunoblotting in Control (untreated with Doxy) mammospheres (ESA$^+$-CD44$^+$-CD24$^-$), whereas it was highly reduced upon

induction of SrcDN by Doxy (2 μg/mL). In addition, ALDH1 was not detected in adherent cell population (data not shown). In a way, our result agrees with data shown by Ginestier et al. indicating that ALDH1 defines a more exclusive subgroup of BCSCs (ESA$^+$-CD44$^+$-CD24$^-$-ALDH1$^+$) of mammospheres, which forms tumors in immunodeficient mice very efficiently (Cell Stem Cell **2007**, 1:555-67, Figure 6E).

Figure 2:

Figure 2A, possibly due to sample size or heterogeneity in the mammospheres, without seeing the full error bars and with the statistical significance being relatively large, this data is not very strong. It is also unclear what the bar and the error bar show. mean or median? SD or SEM?

Sphere Formation Efficiency determination presented in Fig 2A represented the mean (columns) -/+ Standard deviation of the means (bars) of five independent experiments, all of them carried out at least in triplicates. In the revised version of the manuscript we added a new Supplementary S2 Fig, where we presented a Table containing the data of 5 independent experiments carried out for the 3 consecutive generations of mammospheres derived from MCF7-Tet-On-SrcDN and its graphic representations. As observed, there is an increased number of mammospheres, indicating an enrichment of the CD24$^-$ cells population forming mammospheres.

In Figure 2B, the quantitation for the Nanog and Oct3/4 Western blots were the same, but the blot for Oct3/4 was far less apparent compared to Nanog, especially if there were compared to the same β-Actin loading control.

What we are showing in the quantification of Nanog and Oct3/4 are the relative ratios of Nanog/ β-Actin and Oct3/4 / β-Actin considering in each case Control (-Doxy) as 1. However, if we look at the absolute values in the Supplementary Information/Quantification of immunoblots, we observed that the values are different, but the ratios referring to Control as 1 in each case are the same. To be clearer, we have included in the Supplementary Material the full image of all the immunoblots, and the quantification of every band.

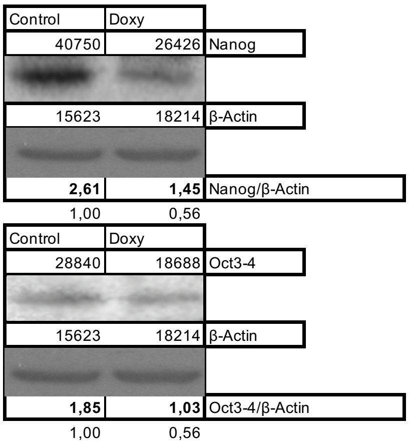

We know that some images of some immunoblots are not perfect. Sometimes antibodies, such as those for Nanog and Oct3/4, are far from been excellent. We have checked 3 different antibodies for each one of these markers, and use the best of them. Nevertheless, we have to admit that these are not good antibodies.

Figure 3:

1. A-C. As in Figure 2, the lack of full error bars and small sample size makes the data less convincing, especially because each graph has the same inferred statistical significance from very different measurements. Again, it is unclear what the error bars represent.

The graphics presented in panels A-C of Fig 3 represent the mean (columns) -/+ Standard Deviation (bars) of the means of 3 independent experiments performed in triplicates Thanks to your comments, we noticed a mistake with the significance in the graph of glucose consumption: it resulted to be less significant. So, we have corrected the error and replaced the old value (***) with the new one (**). We have included now, in the Supplementary Materials, the “pdf” conversion of the final excel document containing the results of the graphs in the Fig 3.

2. In Figure 3D, the blots for Glut-1 and pY10-LDHA, and LDHA are not as well defined as the others, although it could be due to the antibodies. The quantitative decreases in Glut-1, HK2, and pY10-LDHA/LDHA are not very significant and it is unclear whether such level of a decrease can cause changes in glycolytic activity/output. There are no functional validation or rescue experiments done to link the protein level changes to the glycolysis changes.

First at all, as Reviewer indicates, antibodies are not always as good as desired. Nevertheless, we observed a reduction in the level of expression of Glut-1, HK2, LDHA, and pY10-LDHA, proteins that are involved in the control of Glycolysis. These reductions are associated with decreased of glucose transport, and enzymatic activities of hexokinase, pyruvate kinase, and lactate dehydrogenase. These results are consistent with others published (references 18-20). In addition, we added in the revised manuscript a new supplementary figure (new S6 Fig) regarding another aspect of glycolysis control complexity, the effect of HIF-1 transcription factor in the control of HK2 and LDHA expression. As observed by qRT-PCR, SrcDN induction in mammospheres significantly reduced mRNA-HIF-1 expression, concordantly with reduced expression and enzymatic activity of HK2 and LDHA. Since there is a considerable network of glycolysis control, functional rescue experiments would be equally complex to address. We believe that all together these results are consistent and agree with the scientific literature.
